# Gender-biased clustering of attitudes towards physical intimate partner violence: A social network analysis in south-central Ethiopia

**DOI:** 10.1093/pnasnexus/pgaf282

**Published:** 2025-09-03

**Authors:** Mhairi A Gibson, Eshetu Gurmu, Alexandra Alvergne, Daniel Redhead, Sarah Myers

**Affiliations:** Department of Anthropology and Archaeology, University of Bristol, 43 Woodland Road, Bristol BS8 IUU, United Kingdom; Center for Gender and Population Studies, Institute of Social and Economic Research, Addis Ababa University, Addis Ababa 1176, Ethiopia; ISEM, University of Montpellier, CNRS, IRD, EPHE, Montpellier, France; Department of Sociology, University of Groningen, Grote Rozenstraat 31, 9712 TG, Groningen, The Netherlands; Interuniversity Center for Social Sciences Theory and Methodology, University of Groningen, Grote Rozenstraat 31, 9712 TG, Groningen, The Netherlands; Department of Human Behavior, Ecology and Culture, Max Planck Institute for Evolutionary Anthropology, Deutscher Platz 6, 04103 Leipzig, Germany; Department of Anthropology and Archaeology, University of Bristol, 43 Woodland Road, Bristol BS8 IUU, United Kingdom; BirthRites Lise Meitner Research Group, Max Planck Institute for Evolutionary Anthropology, Leipzig 04103, Germany

**Keywords:** intimate partner violence, social norms, social influence, cultural evolution, ALAAM model

## Abstract

Changing social norms, shared beliefs about what is acceptable, is a key focus of global health campaigns aimed at ending intimate partner violence against women (IPVAW). In Ethiopia, it is estimated that one in four women have been assaulted by a male partner, and over half the population hold attitudes supportive of this form of violence. To date, efforts to change people's attitudes towards IPVAW have been hindered by uncertainty over how social norms are acquired or learned. Here, we consider whether people's acceptance of IPVAW is maintained through social influence or “contagion,” using large-scale sociocentric social network data from 5,163 Arsi Oromo farmers in south-central Ethiopia. Bayesian analyses reveal that IPVAW attitudes cluster within social networks. People are more likely to accept IPVAW if the people they chat to, respect, or live with do too. However, exploration of the relationships between social ties indicates that this effect is gender stratified, i.e. driven by same-gender connections. Meanwhile, having IPVAW-accepting social ties of the opposite gender is predictive of a person rejecting IPVAW. Our results indicate that transmission paths may exist among social ties of the same gender: between friends and neighbors, from key respected community figures, and within and beyond households. This suggests that IPVAW prevention interventions that seek to target men *and* women, including key respected community figures of each gender, will be most effective in reducing the acceptability of IPVAW and thus eradicating this form of violence.

Significance Statement A key priority for global campaigns seeking to end intimate partner violence against women (IPVAW) is to change the social norms that underpin its acceptability. However, little is known about how these norms are shaped and socially transmitted. Here, we use social network data and cutting-edge modeling techniques to reveal how social ties are important in predicting IPVAW acceptance among south-central Ethiopian farmers. We identify that IPVAW-acceptance clusters within social networks, indicating that support for IPVAW may be maintained by social influence or “contagion.” Crucially, clustering occurs among social ties of the same gender, within and beyond the household. This indicates that interventions targeting both men’s *and* women's networks will be the most effective in eradicating IPVAW acceptability and behavior.

## Introduction

Intimate partner violence (IPV) is a human rights violation and serious global public health concern (1,). Despite long-standing efforts to eliminate IPV perpetrated by men against women (IPVAW), the most common form of IPV worldwide, it is estimated that one in three women alive today have been assaulted by a male partner (2), and in many countries, this form of violence remains widely tolerated (3)). For example, in Ethiopia, it is estimated that one in four women have been assaulted by a male partner (4) and over half the population hold attitudes supportive of this form of violence (5, 6).

Policymakers seeking to end IPVAW have become increasingly focused on changing social norms (7–10), i.e. the shared beliefs about what is acceptable behavior (11). Various social norms relating to gender roles and violence have been linked to IPVAW; this includes explicit acceptance of IPVAW, which is predictive of IPVAW perpetration (12) and our focus here. Social norms are acquired or “transmitted” through social learning, and norm change interventions seek to change the behavior of individuals by exposing them to alternative values, beliefs, or attitudes of a reference group or person (13). However, simple exposure may not be enough to shift social norms. Cultural evolutionary research has formalized a wide array of biases in social learning, which lead to variation in people’s social influence on, and/or susceptibility to, others (14–17); thus emphasizing the importance of understanding the transmission dynamics of a given norm when designing interventions (18). Examples of successful health interventions that have harnessed social transmission biases to change behavior are still relatively few, but include the use of peer-led influence to reduce substance use among UK adolescents (19) and to increase HIV self-testing among hard-to-reach Kenyan men (20); and the introduction of public declarations to alter the perception of majority behavior to accelerate female genital mutilation/cutting abandonment in Senegal (21). Efforts to design effective norm change interventions to reduce the acceptability, and thus occurrence, of violence towards women by their male intimate partners have for the most part been hindered by uncertainty over how IPVAW norms are shaped and socially transmitted due to a lack of empirical data (22).

Limited knowledge exists on how social interaction within networked communities influences whether individuals perceive IPVAW to be acceptable or not, a key element in understanding norm transmission. This is due to the challenge of obtaining suitable social network data to test for signs of social learning or influence (23). Of the few published studies considering how social interactions within networks may influence the acceptability of IPVAW (24–27), some have focused specifically on understanding the networks of men (26, 27), thus ignoring women's roles in shaping community IPVAW norms. Those that have included women find variability between contexts in the role that women appear to play in IPVAW transmission dynamics, though it is unclear if this is due to differences in sociocultural setting or study methodology (24, 25). No studies to date have described potential IPVAW norm transmission pathways within distinct types of social interaction (i.e. within distinct social networks). Extant studies have relied on aggregate network data, making it unclear which social relationships are more important in the transmission of norms. Further, findings from earlier network studies may have suffered from statistical confounding due to an inability to account for nonindependence of individual outcomes within network data (28, 29), something recent methodological advances have highlighted and addressed (28, 30–32). These techniques are yet to be harnessed in relation to IPVAW. In summary, there remains limited understanding of the extent to which IPVAW acceptance may be acquired through social connections and how social transmission varies across contexts among different kinds of people and relationships within communities (33). This knowledge is important, as social norms can only be exploited by policymakers if key individuals or key networks are identified to initiate norm change cascades.

Here, we seek to advance our understanding of the transmission of social norms that underpin people's acceptance of IPVAW in a rural Arsi Oromo farming community in south-central Ethiopia. Our earlier work with the community suggests that up to a third of adults consider “wife beating” to be acceptable, most notably in instances where women transgress expected norms regarding their household roles and responsibilities (34). This rate of acceptance is similar to that reported for Oromia, the most populous region of Ethiopia (4). Using unique, large-scale cross-sectional sociocentric social network data mapping ties among 5,163 women and men, we explore the distribution of self-reported IPVAW acceptance and perceptions of acceptance among 92.6% of residents of nine neighboring villages (using protocols described in (35)). Further details on sampling can be found in the “Materials and methods” section at the end of the manuscript. The full survey can be found in SI File S3. We explore the composition and structure of three distinct social networks to establish whether certain people and/or social relationships within the community appear more important in shaping norms. For example, we explore the IPVAW acceptance of people holding key positions of respect or power within the community (e.g. religious leaders and kebele/community leaders) and whether they are more centrally positioned within two of the networks, thus better able to control the flow/spread of information. We then use cutting-edge Bayesian statistical models developed to quantify dependencies between individuals in cross-sectional data (28, 35), i.e. the extent to which IPVAW attitudes are predicted by the views of social partners, an indication that IPVAW acceptance *may be* transmitting via social influence or “contagion.”

We assess signals of contagion, as indicated by IPVAW attitude clustering, within three distinct social networks to explore the importance of social ties across distinct types of interaction: community-wide chatting networks, networks of who people respect, and also, networks based on household membership. By including a social network where people are tied by household membership, we are better able to disentangle the effects of social influence from social selection, i.e. the clustering of people based on shared attitudes or other homophilic characteristics, which is a particular problem for cross-sectional network data (30). For example, while shared attitudes towards IPVAW may play a role in the formation of marriage ties (and so household membership), the clustering of IPVAW acceptance within ties between other household members, e.g. parents and children and among siblings, is unlikely to reflect social selection.

Further, by comparing the “household-members” network and its composition with the chatting and respect networks, previously shown to minimally overlap (35), we can also infer whether households, or individuals, are key units for intervention. A previous social network study has argued that IPV interventions that target households, rather than individuals, could be more effective in changing norms (24). However, more recent modeling has argued that information may spread differently within and beyond the household (36). Within the chatting and respect networks, we explore whether there appears to be kinship-biased contagion, and in all three networks, we assess gendered biases (37). This approach was designed to allow us to identify whether there are individuals and groups whose social influence may be key to ending this form of violence towards Arsi Oromo women and whether there are particular relationship types that are promising intervention targets.

## Results

**Fig. 1.**
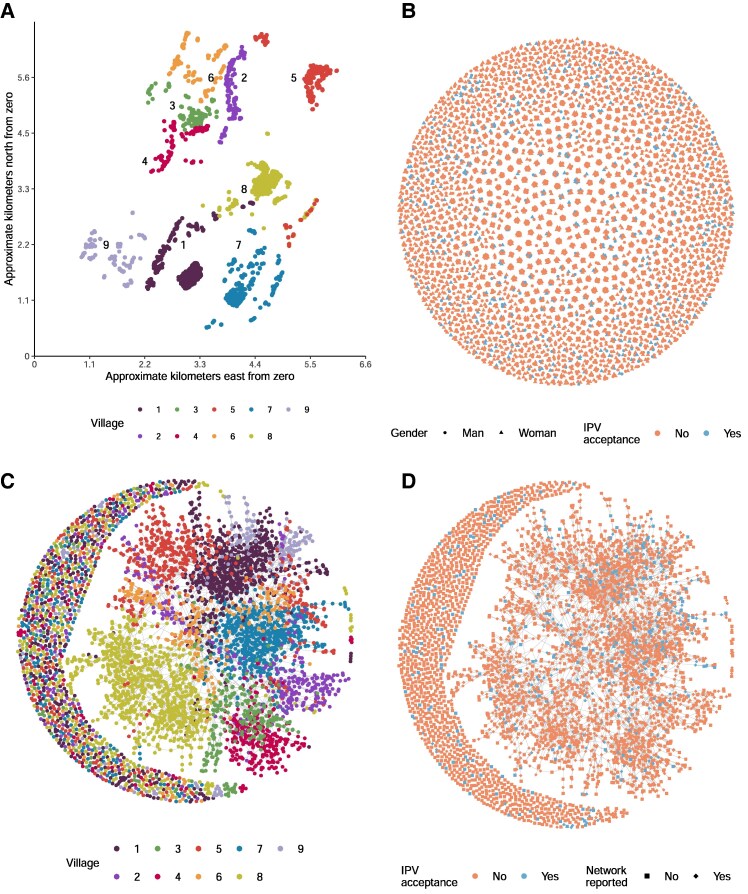
Graph of A) the GPS location of censused households colored by village, B) people (*n* = 5,163) clustered by household co-residency, colored by IPVAW (non)acceptance, and shaped by gender, C) the chatting network with people colored by village, D) the chatting network with nodes colored by IPVAW (non)acceptance and shaped by whether or not they were asked to report their network ties.

A total of 5,163 adults and young people aged 15 years and over (50.2% women or adolescent girls) resident in nine neighboring kebele zones or “villages” were included in the survey and final analyses (see Fig. 1A for the relative geographic distribution of households censused and the Methods for details of sampling and data collection). Villages show some variation demographically; for a full breakdown, see Table S1 in SI File S1 and in (35). The age profile was similar across villages, with median age ranging from 28 (village 2) to 35 years (village 9). Most adults in all villages received no or only partial primary education at similar rates, while the percentage of those receiving secondary education or beyond ranged from 11.7% (village 3) to 22.7% (village 7), and most held no community role.

### Sample IPVAW acceptance

**Fig. 2.**
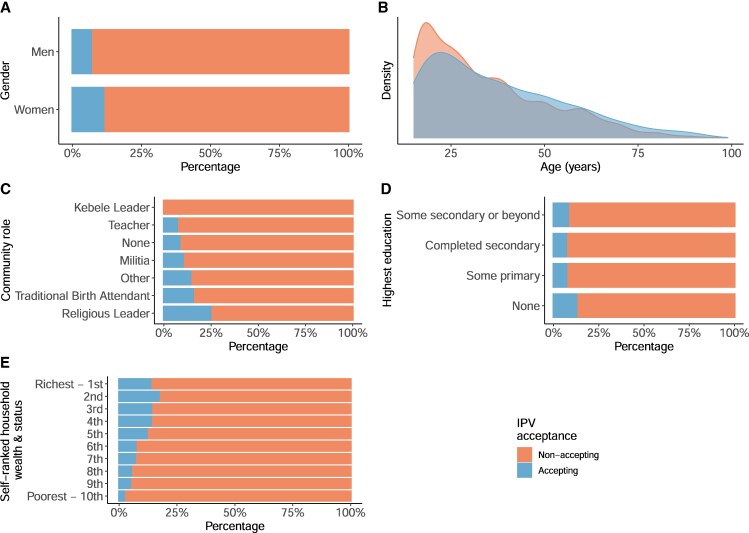
The distribution of IPVAW acceptance by A) gender, B) age, C) community role, D) education in completed years, and E) self-ranked household wealth and status.

Overall, only 9.7% of the full sample self-reported IPVAW acceptance—agreeing with the statement that it is acceptable for a husband to sometimes beat a wife. These rates are lower than those directly reported in our previous survey in a neighboring Arsi Oromo community in 2017 (20%) (34); likely a continuation of a secular trend reported across the entire region of Oromia, dropping 50 percentage points between the 2012 and 2016 Demographic and Health Surveys (DHS) survey (4, 38). By gender, 11.9% of women and 7.4% of men reported that IPVAW was acceptable (Fig. 2A). These gender disparities are in line with previous studies, which indicate that men are more inclined to give socially desirable answers, underreporting their support for IPVAW, due to the social stigma and/or possible legal implications (34, 39). Across the villages, self-reported IPVAW acceptance ranged from 5.3 to 14.8% (Table S1). IPVAW acceptance also showed variation by other demographic metrics, being more common among those who were older (Fig. 2B), held community roles (excepting teachers and kebele/community leaders; Fig. 2C), received no education (Fig. 2D), and perceived their household to be in the richest half of their village (Fig. 2E). In this context older age, wealth, and lower levels of educational attainment all co-vary (Figs. S1–S3) due to new opportunities for education but declining levels of resources (land/livestock) among younger adults (40).

### Perceptions of IPVAW among village neighbors

The majority of respondents (67.8%) reported that some men living in their village thought that “wife beating” was acceptable. However, they typically did not think that the proportion of men supporting it was high: 40% reported that >10% of men thought it acceptable, and <2% that >50% of men did. Perceived acceptance rates of IPVAW among women in the community were reported as being even lower. The majority of people (58%) reported that no women in their village accepted this form of violence. For those people indicating that some women in their village did accept IPVAW, only 16.6% thought that >10% of women did, and <0.5% thought that >50% of women did. Those people who reported accepting IPVAW themselves were more likely to report others in their village as being accepting of IPVAW, compared with those who thought it unacceptable. This could indicate a general tendency for norm misperception in this group (41) and/or underreporting among those who downplayed their own support (and that of others) for IPVAW when questioned directly (see “Discussion” section). This may also simply reflect an availability heuristic: people within social clusters misjudging the prevalence of attitudes in the overall village, as they are oversampling their own social ties (who share their views). Those who accepted IPVAW reported perceiving there were higher levels of acceptance among other men than other women in the community. However, they typically did not think >50% of their village were accepting of it (14% for IPVAW-supporting men and 5% for IPVAW-supporting women). For a breakdown of expectations by village, see Table S1.

### Structure of social networks

**Fig. 3.**
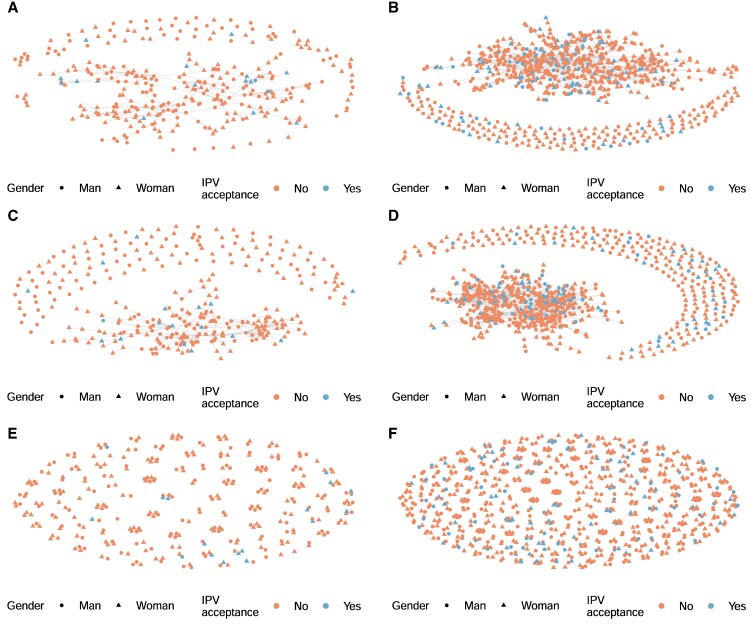
Graphs of A and B) the chatting, C and D) respect, and E and F) household-member networks for the villages with the lowest (A, C, E) and highest (B, D, F) percentage of IPVAW acceptance, with people colored by IPVAW (non)acceptance and shaped by gender.

The structural characteristics of the three networks (chatting, respect, and household-members networks) are reported in Table 1; the household-members and chatting networks are plotted in Fig. 1B and C-D respectively (the respect network can be seen in Fig. S4). The networks show very low density and low reciprocity and transitivity (Table 1). This is partially an artifact of viewing the network at the cross-village level. The chatting and respect networks have been previously reported at the village level (see the Supporting Information of (35), which shows that density, reciprocity, and transitivity per village increase to some degree). While the majority of ties are among village members, a substantial portion of ties occur across village boundaries (as seen in Fig. 1C). Of identified ties, 42% of men's and 46% of women's chatting ties and 38% of men's and 42% of women's respect ties are with people in another village (35). As such, we conduct our analyses at the cross-village level. Visual inspection of IPVAW acceptance clustering *within* villages appears qualitatively similar across chatting and respect networks in the villages. Within-household clustering appears more varied in terms of the proportion of households with multiple IPVAW-accepting individuals and the gender composition where this occurs. Figure 3 shows within-village ties for the villages with the lowest (village 4) and highest (village 7) percentage acceptance of IPVAW acceptance. For the other villages, see Figs. S5–S25.

**Table 1.** Structural characteristics at the network-level.

Characteristic	Chatting	Respect	Household members
Network type	Directed	Directed	Undirected
Ties (*n*)	7,462	6,804	5,748
Kin-only ties (%)^a^	61.3	51.6	—
Same-gender ties (%)	80.2	62.1	37.5
Mean out-degree	1.4	1.3	2.2
Range out-degree	0–11	0–8	0–8
Mean in-degree	1.4	1.3	2.2
Range in-degree	0–9	0–176	0–8
Density	0.0003	0.0003	0.0004
Reciprocity	0.183	0.029	1
Transitivity	0.203	0.067	0.997
Isolates	1,071	1,862	305

Number of ties reflects the total number of relationships held with the 5,163 survey respondents, either as reported by the 2,545 network interviewees (chatting and respect) or calculated based on co-residency (household members). For the interpretation of density, reciprocity, and transitivity, see the “Materials and methods” section. The number of isolates reflects the number of individuals who were either not named in response to the name generator, reported an entirely unidentified network, or were the only adult member of the household found to interview.

^a^It is not possible to estimate the percentage of ties between related individuals within households but given the nature of the network it can be assumed to approach 100%.

### Composition of social networks

Kin were the largest category of social tie being reported across the chatting and respect networks; however, nonkin—which include friends and neighbors—were nominated almost as frequently as figures of respect, accounting for 48% of social ties. There were minimal differences in the proportion of kin versus nonkin nominated by people according to their IPVAW (non)acceptance (Fig. S26). A more detailed breakdown of the composition of social networks by gender and IPVAW acceptance can be found in Figs. S27–S34. These figures reveal that the composition of men's reported social networks is heavily skewed towards other men (Figs. S27 and S31), who most frequently fall into the categories of paternal kin, particularly brothers, and friend/neighbors. Further, there is an indication that IPVAW-accepting men were more likely to nominate other men classed as friends/neighbors in both chatting and respect networks than IPVAW-rejecting men. In terms of men's social ties with women, men almost exclusively nominated only their mothers and wives (Figs. S28 and S32). Women's reported social networks were more diverse (Figs. S29, S30, S33, and S34). Their networks were more dominated by friends/neighbors, and they also report frequently chatting to husbands and sons (Fig. S30); however, mothers-in-law were also often nominated in respect networks (Fig. S33), husbands’ brothers received a moderate proportion of nominations in both chatting and respect networks, and unrelated men received a moderate share of chatting and frequent respect nominations (Fig. S34). Given women's reports, this indicates men underreport at least their chatting interactions with women (one of the many common biases in network measurements: (42)). This may reflect differences in the value placed on mixed-gender interactions in this patriarchal society (the implications of this are outlined in the Discussion). In terms of the particular people named within the chatting and respect networks and identified as household members, there was little overlap, indicating the three networks are largely distinct from each other: the Jaccard similarity coefficient (where 1 indicates complete overlap and 0 none) for chatting-respect overlap was 0.138; for chatting-household members, it was 0.013; and for respect-household members, it was 0.006.

In terms of the network centrality, an indicator of control over information transmission, there was little to distinguish between those who do and do not accept IPVAW, though the upper range of receiving respect nominations was higher among IPVAW nonaccepting individuals (176 vs. 44; Table S3). Those holding certain roles in the community, by some metrics, were more centrally positioned within community-wide networks (Table S3), suggesting they may play a greater role in social transmission. Within the chatting network, Kebele leaders *received* and *made* slightly more nominations on average than those with no role, as did religious leaders, militia, and those classed as having “other” roles. Kebele leaders had a higher average and upper range of vertex betweenness, indicating they are more likely to lie on the path between two unconnected individuals in the network, a proxy for their having greater control over the flow of information between people. Kebele leaders, religious leaders, militia, and those with “other” roles also had higher average harmonic centrality scores in each network, indicating they are closer to all other people across the communities and thus may be more influential. In terms of respect, apart from traditional birth attendants and teachers, those with community roles on average also *received*, but did not *make*, more nominations. However, any greater influence of role holders is not absolute; the lower bound of all centrality measure ranges, across role categories, was zero and the upper bound of those with no community role came close to or exceeded those of role holders (e.g. the in-degree range of religious leaders was 0–176, and for those with no role, it was 0–117).

### IPVAW shows signals of maintenance by social influence

**Fig. 4.**
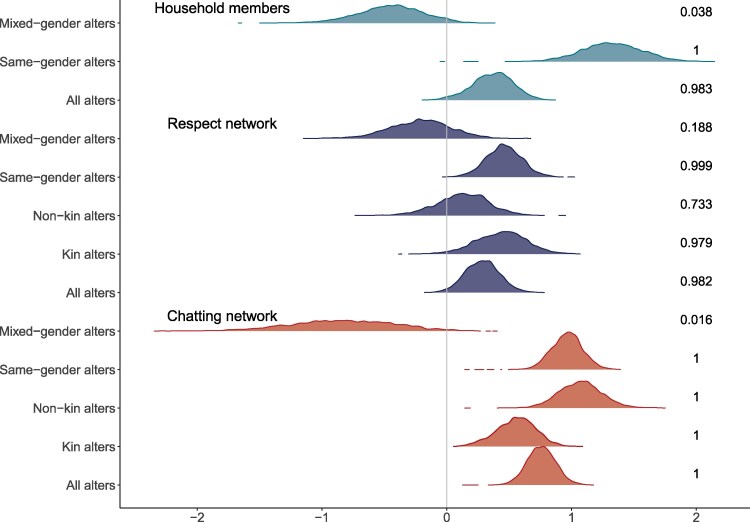
Posterior distributions from ALAAM models estimating contagion of IPVAW acceptance. A positively skewed posterior distribution (i.e. >0) indicates an individual is more likely to report accepting IPVAW if they are socially connected to another person who also accepts IPVAW. Numbers on the right on the plot indicate the percentage of the posterior in favor of positive contagion.

Bayesian autologistic actor-attribute models (ALAAMs) are used to assess signals of IPVAW-acceptance “contagion,” an indicator of potential social influence, within different networks. Here, a posterior distribution falling mainly above zero indicates a person is more likely to be IPVAW accepting if they are directly connected to another person (i.e. an alter) who accepts IPVAW. The models are all adjusted for gender, education, self-ranked household wealth and status, village, and number of nominations made (out-degree). Chatting and respect network models are also adjusted for the number of nominations received (in-degree; for details, see the “Materials and methods” section). For full model results, see SI File S2. Across networks, we find a positive direct contagion signal (Fig. 4) with a person predicted to be more likely to be IPVAW accepting if they reported that they chatted to, respected, or lived with another person who accepts IPVAW. Among the chatting network, the direct contagion signal appears stronger among nonkin alters, while kin have a stronger effect in the respect network. Across chatting, respect, and co-residency alters, positive direct contagion was indicated among same-gender alters. Exploring the distribution of ties across possible combinations of ego-alter × IPVAW (non)acceptance indicates that both genders contribute to this effect within households (Table S4); however, in the chatting and respect networks, it is driven by woman–woman ties (Table S5).

The posterior distributions for mixed-gender alters were all negatively skewed, indicating a person was *less* likely to accept IPVAW if they were directly socially tied to or lived with a person of the opposite gender who accepted IPVAW, suggesting that if there is transmission of acceptance, it does not typically pass through mixed-gender routes. The very wide distributions for chatting and respect are reflective of the strongly gendered nature of nominations reducing the available sample size, while within household, though ties are more likely to be mixed-gender, the sample sizes are somewhat smaller (Table 1). Exploring the distribution of ties across possible combinations of ego-alter × IPVAW (non)acceptance again indicates that both genders contribute to this effect within households (Table S4); while in the chatting and respect networks, it is being contributed to by both genders but is stronger when the ego is a woman (Table S6).

Post hoc exploration of ties between the subsample of people who could both receive and send nominations in the chatting and respect networks, adds further descriptive information regarding the clustering of IPVAW acceptance. Edge-directionality tests, assessing the presence of asymmetry in the likelihood of attribute sharing dependent on whether a tie is outgoing or incoming, can assist in drawing inferences between social influence and social selection. This relies on the assumption that though influence may produce symmetrical estimates (e.g. when influence is reciprocal or works in conjunction with selection), selection is not expected to produce asymmetrical ones (37, 43). We find no evidence of asymmetry in the direct contagion signal, dependent on the direction of the tie (Fig. S35), meaning we cannot rule out the action of social selection in underpinning our findings on these grounds. On the other hand, there are also relatively weak signals of more complex forms of contagion (Fig. S35), which possibly favor social influence. While in longitudinal data the results of such tests may be taken as evidence regarding the causal effect of social influence, such interpretation has been cautioned against when applying to cross-sectional data (29).

## Discussion

We set out to describe the social distribution of attitudes towards, and perceptions of, IPV against women (IPVAW) within nine Arsi Oromo villages in south-central Ethiopia. Using self-report data from 5,163 adults, we find that ∼10% of people believed “wife beating” was acceptable, though this number is likely to be an underestimate due to reporting biases (further details are outlined below). By analyzing sociocentric social network data, we identify that a person living in this community is more likely to accept IPVAW if they are socially connected to another person who accepts it. This is suggestive that attitudes towards IPVAW could be transmitting via social influence. A unique feature of this work is that we have been able to explore signals of social contagion across three different kinds of social relationships: among household co-residents, people who spend time chatting together, and among people in respectful relationships. We have also been able to explore other biases in potential social transmission, e.g. due to gender, kinship, and/or role in the community, as described below.

While causal inference remains speculative in cross-sectional data, our analyses identify signals of social contagion, which are consistent with social influence acting within the chatting and respect networks irrespective of the kinship nature of the tie. In each network we find signals of *positive* contagion when social ties are the same gender (Fig. 4); however, we find signals of *negative* contagion when social ties are between opposite-gender individuals (Fig. 4). This indicates that IPVAW acceptance is socially clustered and may be transmitting within but not between genders, thus highlighting the importance of considering the role of Arsi Oromo women (as well as men) in maintaining social norms harmful to women. Our findings also differ from Shakya et al.'s social network study (24), which identified that opposite-gender relationships are strongly associated with IPVAW acceptance in rural Honduras, thus emphasizing the need for policymakers to consider local context-specific information when designing interventions to change IPVAW norms and behaviors.

Additional exploratory analyses reveal how a negative contagion effect could be operating within Arsi Oromo households. We found that both husbands and wives were slightly less likely to report IPVAW acceptance if their spouse reported acceptance. Where daughters reported IPVAW acceptance, 39% of their co-resident mothers did too (compared with 0% of their fathers). The pattern was weaker among men; nevertheless, among IPVAW-accepting sons 17% of their fathers were accepting (compared with 8% of their mothers; Table S4). This underlies the clear opposing contagion signals seen within mixed- vs. same-gender household ties. The origin of the tendency for opposing views between mixed-gender social ties is unclear, but a possible explanation is that IPVAW-accepting men may be more likely to assault their wives (12), and being assaulted causes a portion of previously IPVAW-accepting wives to change their attitude, with contagion effects then occurring within and beyond the household. While in high-income contexts, experience of domestic violence is often associated with victims justifying their partner's actions (44), such reactions may vary contextually. Indeed, using longitudinal data from rural India, Shakya et al. (45) have shown that women are more likely to reject IPVAW after experiencing violence from an intimate partner. That we find signals indicative of possible gender-biased social transmission suggests that if Arsi Oromo women victims change their attitudes towards IPVAW, they may also go on to influence other women in their social networks (Figs. S29 and S33). They may no longer act as a source of influence in IPVAW acceptance and may even exert influence in spreading nonacceptance attitudes among other women in the community (Tables S4 and S5). The same logic applies to when men change their attitudes, subsequently influencing other men, though it is less clear how acceptance in a wife would trigger a husband to become unaccepting (Figs. S27 and S31, Tables S4 and S5).

Unlike other social network studies, we find no clear indication of social clustering being solely biased towards closely related (46) and/or co-resident people (24), indicating that individuals, not households, should be targeted for interventions. Further, our social network data indicate that same-gender friends and neighbors are the most frequently nominated social ties for both men and women (Figs. S27–S34), which could lend support to theoretical models that predict that focusing on friendship-nomination targeting is the most effective way to enhance the adoption and spread of beneficial interventions (33, 47).

Our results also suggest that key community role holders and respected figures are important in the distribution of IPVAW acceptance. Religious leaders, who are typically men, hold some of the highest rates of IPVAW acceptance within the community (Fig. 2, Table S2) and, by some metrics, are more central within community-wide networks (Table S3). Their higher centrality suggests that if IPVAW acceptance is socially transmitted, they may play a greater role in transmission to other men, though seemingly not to women. Mothers-in-law, frequently nominated as figures of respect by women, are a likely candidate for specific women outside the home who may be influencing daughters-in-law tolerance of IPVAW in a similar way (Fig. S33). Any influence of these respected community figures on IPVAW acceptance is likely to reflect their knowledge of and authority over upholding gender-specific norms, expected household roles, and responsibilities. Women's transgression of these gender norms is frequently used to justify IPV perpetrated by Arsi Oromo men (34). Our results also suggest that kebele/community leaders are the most consistently central, and thus, influential role holders within community-wide networks (Table S3); they are also among the people least likely to endorse IPVAW (Fig. 2, Table S2). This could indicate that they have a key role in reducing acceptance of IPVAW within the community or that they are more inclined to underreport their support for it. Both scenarios are possible due to kebele leaders' greater awareness of IPV's illegality, as well as their responsibility for enforcing these federal laws within the community. While community leaders are clearly influential, further studies are needed to disentangle these possible effects to inform future interventions.

There are some limitations that should be considered when evaluating the results of this study. One limitation is that we have not been able to statistically disentangle the effects of social influence (or learning) from the effects of social selection for homophily (selecting social partners based on shared attitudes to either IPVAW or other shared attributes correlated with acceptance) or simularity due to shared exposure. Although we cannot rule out social selection without longitudinal data (and post hoc edge-directionality tests tentatively favor it), our study has been designed to mitigate against this limitation. Specifically, we compare attitudes with IPVAW within households, where ties are based on co-residency and the clustering of attitudes is unlikely to reflect social selection on the basis of shared IPVAW acceptance. While it is possible that shared attitudes contribute to selection among husbands and wives, parent–child and sibling selection within households is improbable. Further, the gendered nature of clustering identified within households makes shared exposure less probable. Social influence and selection are plausibly both at play; nevertheless, the fact that we observe similar attitudinal patterns within households and across networks gives us confidence to suggest that social influence makes a greater contribution to our contagion estimates than social selection.

A further potential limitation with our study is the likelihood of two areas of underreporting in our data. First, we used direct questioning to gauge IPVAW acceptance, which may mean that the 9.7% IPVAW acceptance found is an underestimate. Using indirect questioning techniques in 2017, we found that some people in a neighboring Arsi Oromo community may be inclined to conceal their support for “wife beating” when questioned directly (34), a tendency that has been shown to be greater among men (39), due to social stigma and potential legal implications. Although indirect questioning techniques provide respondents with greater privacy, resulting in more reliable responses, we did not use them here, as they produce population-level estimates—so are incompatible with social network analysis which requires individual-level data. That 40% of participants in our current sample thought that at least 20% of men in their village accepted “wife beating,” is a further indication that the level of male acceptance of IPVAW reported here (7.4%) is an underestimation. However, this supposition is against a background in which levels of IPVAW acceptance have been declining; our previous 2017 work recorded directly reported acceptance levels of 20% (34), close to the regional average of 28% measured by the DHS (4). Second, it is possible that people may also be inclined to mis-report their social networks (42). The absence of mixed-gender ties in men's networks, despite their presence in the networks of women, indicates that men may be underreporting their interactions with women (Figs. S27–S34). This may reflect gender differences in the value placed on mixed-gender interactions, with men inclined to disregard or forget their interactions with women in this patriarchal and patrilocal setting. This presents two possible scenarios. If the IPVAW views of women are similarly devalued, this should not greatly impact our ability to detect women's social influence in men's networks. However, if we are missing ties through which women do exert influence on men, then our estimates of mixed-gender contagion are likely to be underestimates. The household-member networks we use based on co-residency are, however, free from these potential reporting biases. That our results regarding mixed-gender ties are very similar across all three networks, including the household network, suggests the former scenario is most likely (i.e. woman's influence on men's IPVAW attitudes is minimal). These findings also highlight the complexity of studying social learning in real-world settings, in which understanding is constrained by people's perceptions of the relationships through which transmission occurs (48).

Overall, these findings indicate that IPVAW-acceptance clusters between people of the same gender, within several different kinds of social relationship. This suggests that IPVAW attitudes may be transmitting between same-gender friends and neighbors; obliquely from respected figures within the community, for example, from religious leaders to other men and mothers-in-law to daughters-in-law; and within households between same-gender siblings, fathers and sons, and mothers and daughters. These results imply that interventions seeking to reduce the acceptance of IPVAW among Arsi Oromo communities should target both men and women, and that preexisting and widespread interventions targeting men-to-men chatting networks (49) could be usefully adapted to target women-to-women networks too. Further, identifying sites of same-gender interaction (e.g. meetings held by gender-specific civil society organizations like *Iddirs*) could also be one effective way to initiate changes in IPVAW norms and behaviors within these communities.

It is an open question as to how generalizable these findings are. We, however, consider this knowledge to be critical for policymakers seeking to efficiently change attitudes and social norms to reduce IPVAW. Lessons can be taken from the female genital mutilation/cutting literature, where the widespread application of interventions based on the social dynamics documented in one population have had limited success in other contexts (50). The necessary social network data are currently extremely rare. Including ours, the three extant studies from which empirical insights into IPVAW social norm transmission can be drawn identify culturally varying patterns (24, 25). This should serve both as a caution against a “one-size-fits-all” approach to norm change and an impetus to invest in further, in-depth data collection to help protect the women and girls of the future.

## Materials and methods

### Data collection

Data collection took place between 2021 and 2022. Data were collected from nine neighboring administrative kebele zones or “villages,” within the same Woreda, or “district” in the Arsi Zone, southern Oromia, south-central Ethiopia. This population was selected as previous survey data indicated that IPVAW-acceptance rates were similar to those of Oromia, the most populous region of Ethiopia (4, 34) ), but also due to our long-term (20 years) engagement with the community, ensuring acceptance of questioning on this sensitive topic. Surveys were conducted in Afaan Oromoo by research assistants trained in demographic field survey methods, recruited and trained by Addis Ababa University. Community members were informed of the nature of the research during the weekly village meetings, where they were invited to openly discuss their involvement in the study and to ask questions of the project staff. Informed consent was obtained from each individual participating in the study. Research and ethical approval to undertake the study were granted by the Research and Ethics Committees at the University of Addis Ababa (Ethiopia) and the University of Bristol (United Kingdom). Further details of the research protocols can be found in our previously published paper (35); and details of Arsi Oromo subsistence and marriage practices can be found in (51).

First, a *Household Census* was conducted in 2021, with the aim of visiting each household within the nine villages, identifying all occupants aged 15 years or over (referred to as adults throughout) and assigning them individual ID codes composed of a four-digit household identifier and two-digit individual identifier. Information was collected from 5,578 individuals across 1,949 households. A *Norms and Networks Survey* was subsequently conducted in 2021–2022, with the aim of collecting attitudes towards IPVAW from all individuals identified in the *Household Census*, as well as social network data from 50% of respondents. The full survey can be found in SI File S3. The final sample encompassed 5,163 participants (49.8% men, 50.2% women), and 2,545 (49.3%) of whom reported social network data. Of 54,632 alters (i.e. social ties) named by respondents in response to six network name generator questions (two of which we use here), 41,235 (75.5%) were assigned IDs from the *Household Census* using Levenshtein similarity scores and 39,572 (72.4%) were identified as having participated in the *Norms and Networks Survey* making them eligible for analyses, because we had information on their attitudes to IPVAW. Further details and discussion on how social ties were identified can be found in SI File S1 and (35).

During data collection respondents were randomly assigned to one of two different versions of the survey: version “A” included a social network name generator, and version “B,” which did not. Both versions included questions regarding individuals' sociodemographic background including age, educational attainment, gender, community role, perceived household socioeconomic position, the name of the head of household, and their relationship to them; as well as questions about the acceptability of IPVAW. Respondents receiving version A of the survey were also asked to report their social network connections (up to a maximum of 10) in a number of domains: here, we use chatting and respect nominations, whose solicitation was motivated by our considering them likely candidates as IPVAW attitude transmission paths. For each reported social tie (i.e. alter), their three names (their given name, their father's name, and their paternal grandfather's name) were recorded along with information on their age, gender, relationship to the interviewee, and residence (the same village or elsewhere); these data were used to match alters against censused individuals and assign ID codes (details in SI File S1).

The chatting network was estimated using responses to the name generator “Who do you spend time chatting with?” and the respect network with responses to “Who do you respect and admire?.” The household network was constructed by assigning ties to individuals sharing household ID codes. Ten men and one woman were named as the heads of multiple households, these households were treated separately, with only the head tied to individuals across households (i.e. the head's ties included members of households A and B, whereas nonheads in A were not tied to nonheads in B and vice versa). As respondents were only asked their relationship to the head of household, rather than all household members, it is not possible to accurately assign kinship status to individual household ties; however, as only 33 people reported being a “nonrelative” of the head, it is reasonable to assume nearly all ties were between relatives.

Views on the acceptability of IPVAW were assessed by asking respondents a series of questions about men's attitudes towards their wives. Participants were asked, “Do you think it is acceptable sometimes for a husband to beat his wife?” and to respond by answering “Yes” or “No.” To assess empirical expectations regarding local support for IPVAW, respondents were also asked to report what they thought other people’s views would be within the kebele zone. Using a visual scale from 0 to 100%, with 10% increments, respondents were asked to identify how many men and, then, how many women would find it acceptable sometimes for a husband to beat his wife within their kebele zone.

Educational attainment was operationalized as “none,” “some primary,” “completed primary,” or “some secondary or beyond” (further details on how this was collected can be found in (35)). Self-ranked household wealth and status were obtained using a wealth ranking exercise. Using a visual scale of houses of descending size labeled from one to 10, along with the instruction that one represented the richest 10% and 10 represented the poorest 10% living in their kebele zone, respondents were asked to identify the wealth and status position of their household within their kebele zone.

### Analyses

#### Descriptive statistics

We calculate descriptive statistics for our networks with the *igraph* package (52). For each network, we report: number of ties; the percentage of ties to alters who are also kin (chatting and respect only); the percentage of ties to alters of the same gender; density, i.e. the ratio of actual ties reported to possible ties, reflecting the connectivity of the network; reciprocity, i.e. the proportion of relationships that are mutually reported (though note this estimate is biased downwards by only a portion of the sample reporting their chatting and respect ties); transitivity, i.e. the proportion of ties that form closed triangles within the network, reflecting the clustering of the network (this will be similarly biased downwards), and; the number of isolates, reflecting the number of individuals who were either not named in response to the name generator, reported an entirely unidentified network, or were the only adult member of the household found to interview.

We further report measures of median centrality and their range, dependent on whether individuals were IPVAW accepting or not and on their community role, to infer whether these individuals appear to be of particular influence: in-degree, i.e. the number of nominations received; out-degree, i.e. the number of nominations made; vertex betweenness, which measures the extent to which an individual lies on the path between other individuals in the network, proxying their control over the flow of information between others; and harmonic centrality, which measures how close an individual is to all other individuals in the network and proxies the speed at which information could spread from a given individual to all other individuals. Higher scores in each of these measures indicate greater centrality. As measures of centrality are known to be biased by partial network sampling (53), we do not interpret the resulting values directly; however, to the extent that the networks of pro- and anti-individuals can be assumed to be similarly impacted, their comparison is of interest.

#### Assessing whether IPVAW acceptance is maintained by social influence

As reported in (35), the chatting and respect networks show low overlap in terms of nominations, as indicated by a Jaccard similarity coefficient of 0.138. Additionally, both networks show negligible overlap with the household network: the chatting-household coefficient was 0.013 and the respect-household coefficient 0.006. As such, we model each network separately.

To test for signals of “direct social contagion” of IPVAW acceptance, which are expected if social influence is acting within networks, we focus on alters named as part of the focal individual's chatting network (ties *n* = 7,462) and the network of people they reported respecting (ties *n* = 6,804), as well as household-member networks based on co-residency (ties *n* = 5,748). We run a separate ALAAM for each network type using the *BayesALAAM* function, available as part of *MultivarALAAMalt.R* (28). The outcome variable is a binary measure of whether an individual reported “no” (0) or “yes” (1) to wife beating being sometimes acceptable. From a matrix of ties between all individuals in the sample, the model constructs a *direct contagion* parameter based on the IPVAW-acceptance profiles of alters of a given individual within the matrix; a resulting positively skewed posterior distribution of estimates for direct contagion indicates a person is more likely to be IPVAW accepting if they are directly connected to another person who accepts IPVAW. In addition to contagion, the chatting and respect models are conditioned on the focal individual's out-degree, in-degree, gender, education, self-ranked household wealth and status, and village. The household-member models were the same apart from not containing in-degree (which is the same as out-degree in this network and becomes a proxy for household size), as indicated by our directed acyclic graphs (DAGs) of inferred causal relationships underpinning IPVAW norms (Figs. S36 and S37). We use the minimally informative priors recommended by (28), which ensure that our priors are informative enough to stabilize estimation but do not impose overly strong assumptions on the parameter space. To explore the possibility that contagion occurs differentially depending on *whom* ties are shared with, we also re-run each model assessing same-gender (chatting ties *n* = 5,987, respect ties *n* = 4,228, household ties *n* = 4,314) and mixed-gender alters (chatting ties *n* = 1,475, respect ties *n* = 2,576, household ties *n* = 7,182) and, in the chatting and respect networks, kin-only (chatting ties *n* = 4,574, respect ties *n* = 3,511) and nonkin-only alters (chatting ties *n* = 2,888, respect ties *n* = 3,293).

### Post hoc tests

Though it is not possible with cross-sectional data to conclusively determine that clustering on a given attribute is the result of social influence, rather than the product of social selection for the attribute or otherwise homophily for an unmeasured factor or shared exposure, we also perform a number of post hoc tests to add descriptive information regarding IPVAW-acceptance clustering. Edge-directionality tests, assessing the presence of asymmetry in the likelihood of attribute sharing dependent on whether a tie is outgoing or incoming, have been proposed as a way of evidencing social influence in directed networks (37), on the assumption that though influence may be symmetrical, homophily is not expected to be asymmetrical. While such tests have received strong critique (29), the results nevertheless add descriptive information regarding the clustering of IPVAW acceptance. As our partial network sampling introduces bias by constraining the possibility of some people having outgoing ties, we confine this analysis to just ties between the half of the sample who reported their networks (for the network characteristics of this subsample, see Table S7, and for a detailed comparison with the full sample, see (35)). To implement edge-directionality tests within an ALAAM framework, we run two versions of the chatting and respect models on (i) the originally formatted tie matrices (reflecting outgoing ties) and (ii) the transposed tie matrices (reflecting incoming ties) and compare the resulting direct contagion estimates (43).

Finally, the presence of signals of higher-order contagion within a network, which are theorized as arising from social processes (54), also adds to the descriptive picture of clustering; in longitudinal data their presence would be harder to explain via homophily, though again interpretation in a cross-sectional context relies on strong assumptions (29). We also run variants of the chatting and respect models, including parameters for the additional dependencies supported within *BayesALAAM* allowing for the estimation of “reciprocal,” “indirect,” “closed-indirect,” and “transitive” contagion (for more details, see SI Files S1 and S2).

As each of these models entails the subsetting of the data, we confine these post hoc tests to the analysis of all alters and do not further reduce the sample to look at different types of ties, the analysis of which would likely be underpowered.

SI File S1 contains Tables S1–S7 and Figs. S1–S37. SI File S2 contains the full ALAAM results, and SI File S3 contains the full survey. All network plots were made with *ggnet* (55), and the map of households was made with *ggplot2* (56).

## Supplementary Material

pgaf282_Supplementary_Data

## Data Availability

Data and code for replicating the analyses are available on the Open Science Framework at https://osf.io/mysrk/.

## Preprints

This manuscript was posted on a preprint at: https://osf.io/preprints/osf/57yuf_v2.
