# Supplementary material for: Gender-biased clustering of attitudes towards physical intimate partner violence: A social network analysis in south-central Ethiopia
Source: PNAS Nexus. 2025 Sep 3;4(9):pgaf282. doi: 10.1093/pnasnexus/pgaf282 (PMC12448887; doi:10.1093/pnasnexus/pgaf282)

**Supplementary information file 1: Gender biased clustering of attitudes towards physical intimate partner violence: a social network analysis in south-central Ethiopia**

**Additional Materials and Methods**

*Identifying social ties*

Participants of the *Norms and Networks Survey* and the social ties (i.e. alters) who they named were assigned IDs, where possible, from the *Household Census* using Levenshtein similarity scores in the first instance, and further verified via the cross-checking of additional demographic information collected in both surveys.

Everyone in this community has three names, each of which respondents were asked to report when naming ties, in addition to which respondents were asked to report the tie’s age, gender, relationship to the respondent, and whether they lived in the same village. There are inheritance rules tied to naming, such that a person’s second is the first name of their father, and their third name is the first name of their father’s father; this resulted in there only being a few individuals within our household census that have the same name as someone else censused and in each case they differed by multiple decades in age, preventing misidentification.

However, there are two primary sources of potential error when assigning IDs: Not confining our respondents to naming ties from the same village introduces the possibility of misidentification of named alters. For example, where person 1 and person 3 both live in village 6 and they voluntarily name a person 2, called John Paul Ringo, with similar age, and state he lives in the same village, we can be confident in our conclusion they are naming a) the same person and b) the John Paul Ringo we surveyed in that village. But we cannot guard against a scenario in which person 1 lives in village 5 and names a John Paul Ringo aged 28 from a different village, and person 3 lives in village 4 and names a John Paul Ringo aged 30 from a different village, and there is a John Paul Ringo aged 29 living in village 6 who took part in the survey, leading us to identify as him the individual person 1 and 3 were naming, when in fact one or both were referring to man of this name and approximate age from outside of the study area. However, as only a few people share a name in our census of over 5000 people, the risk of this form of error being frequent is likely to be very low. Nevertheless, increasing the chances of such an error is the fact that age reporting in this context is best taken as an approximation – people might not know the exact age of social ties and self-reporting of own age may also be inaccurate among older individuals – we thus allowed reported ages to ‘match’ if they fell within a range of that recorded in the census (with the permitted range increasing with age).

These issues are of much less concern when it comes to the household members network. Here we were assigning residents to the same household using the survey respondent’s self-reported names, age, gender, village of residence, the name of their household head, their relationship to the head, their level of education and whether they held a community role to same data collected during the census, leaving minimal scope for error. As such, the similar results in the chatting and respect analyses when compared to the household members analysis add confidence that error induced bias in the former is not driving our results. It is rare that data is error free, yet we are sufficiently confident that the noise introduced by misidentification is not to the degree that it would alter direction of our results.

*Post hoc tests*

Using the subsample of data from people who reported their network, we conducted *post hoc* explorations of models with the inclusion of additional dependencies, facilitating the estimation of more complex forms of contagion as enabled by *BayesALAAM*: *reciprocal contagion* captures whether a person is more likely to be IPVAW accepting if they are mutually tied to another respondent who is accepting; *indirect contagion* captures whether a respondent is more likely to be IPVAW accepting if they are indirectly connected to respondents who are accepting; *closed indirect contagion* captures whether a respondent is more likely to be IPVAW accepting if they are both directly and indirectly tied to other respondents who are accepting, and; *transitive contagion* captures whether a respondent is more likely to be IPVAW accepting if they are embedded in triads where the other two members are accepting. The statistical principle of hierarchy necessitates that lower-order effects of a given contagion parameter are modelled along with higher-order effects, thus we made the following stepwise additions to the original direct contagion models: 1) the reciprocal contagion parameter (Σ*_i_*_<_*_j_*Y*_i_*Y*_j_*X*_ij_*X*_ji_*) and the number of reciprocal ties; 2) the indirect contagion parameter (Σ*_i_*Y*_i_*Σ*_i_*X*_ij_* Σ*_k≠i,j_*Y*_k_*X*_jk_*), mixed two-path ties as a measure of brokerage, and the number of indirect ties; 3) the closed indirect parameter (Σ*_i_*Y*_i_*Σ*_i_*X*_ij_* Σ*_k≠i,j_* Y*_k_*X*_ik_*X*_jk_*) and the number of indirect ties excluding those that are also direct ties, and; 4) the transitive contagion parameter (Σ*_i_*Y*_i_*Σ*_i_*X*_ij_*Y*_j_*Σ*_k≠i,j_*Y*_k_*X*_ik_*X*_jk_*) and the number of embedded transitive triads. Though the posterior distributions were wide, the bulk of the reciprocal contagion estimate fell to one side of the null in both the chatting and respect models and so it was also retained in each of the subsequent steps. As indirect contagion is a lower-order effect of both closed indirect and transitive contagion, the parameters added in step 2 were also retained for both steps 3 and 4. The posterior distributions from these models can be seen in Figure S13.

**Table S1** Demographic characteristics by village

|  | | **Village** | | | | | | | | |
| --- | --- | --- | --- | --- | --- | --- | --- | --- | --- | --- |
|  |  | **1** | **2** | **3** | **4** | **5** | **6** | **7** | **8** | **9** |
| **n** | | 1031 | 400 | 316 | 322 | 633 | 305 | 820 | 1122 | 214 |
|  | | Median (IQR) | | | | | | | | |
| **Age (years)** | | 30  (22) | 28 (23.5) | 30 (24.25) | 30 (29.75) | 29  (27) | 30  (23) | 32  (26) | 30  (22) | 35  (24) |
|  | | Percentage | | | | | | | | |
| **IPV-accepting** | | 11.445 | 6.75 | 5.696 | 5.280 | 11.216 | 11.475 | 14.756 | 6.239 | 10.28 |
| **Male** | | 48.788 | 53.081 | 47.196 | 49.684 | 49.287 | 49.250 | 47.205 | 48.852 | 51.341 |
| **Highest education** | None | 24.442 | 27.000 | 27.215 | 24.534 | 23.697 | 27.213 | 21.707 | 22.549 | 27.103 |
|  | Some primary | 48.109 | 48.750 | 49.051 | 50.311 | 50.237 | 45.246 | 42.805 | 44.652 | 46.729 |
|  | Completed primary | 9.893 | 10.500 | 12.025 | 12.422 | 10.742 | 12.459 | 12.805 | 11.854 | 10.280 |
|  | Some secondary or beyond | 17.556 | 13.75 | 11.709 | 12.733 | 15.324 | 15.082 | 22.683 | 20.945 | 15.888 |
| **Community role** | None | 92.629 | 93.250 | 89.241 | 93.478 | 93.049 | 91.475 | 89.634 | 94.474 | 91.589 |
|  | Role | 7.371 | 6.750 | 9.810 | 6.522 | 6.951 | 8.525 | 10.366 | 5.526 | 8.411 |
| **Perceived percentage of men in their village who think wife beating is acceptable** | 0% | 27.449 | 34.75 | 22.152 | 53.106 | 37.599 | 36.066 | 21.22 | 38.948 | 19.159 |
|  | 10% | 33.754 | 30.5 | 17.089 | 32.919 | 31.28 | 20.656 | 31.463 | 19.875 | 29.439 |
|  | 20% | 13.385 | 11.5 | 9.81 | 6.211 | 12.954 | 21.311 | 17.927 | 16.221 | 23.832 |
|  | 30% | 10.669 | 13 | 15.19 | 3.727 | 10.9 | 12.131 | 12.195 | 14.528 | 10.748 |
|  | 40% | 9.02 | 4.5 | 20.886 | 1.863 | 2.844 | 5.246 | 7.561 | 6.15 | 7.477 |
|  | 50% | 4.268 | 3.5 | 11.076 | 1.242 | 3.476 | 3.934 | 7.073 | 3.387 | 7.477 |
|  | 60% | 1.067 | 1.25 | 3.797 | 0.621 | 0.948 | 0 | 2.073 | 0.446 | 0.467 |
|  | 70% | 0.097 | 0.75 | 0 | 0 | 0 | 0 | 0.244 | 0.089 | 0 |
|  | 80% | 0.291 | 0.25 | 0 | 0 | 0 | 0.328 | 0.244 | 0.267 | 0.467 |
|  | 90% | 0 | 0 | 0 | 0 | 0 | 0 | 0 | 0 | 0 |
|  | 100% | 0 | 0 | 0 | 0.311 | 0 | 0.328 | 0 | 0.089 | 0.935 |
| **Perceived percentage of women in their village who think wife beating is acceptable** | 0% | 48.4 | 70 | 53.481 | 72.05 | 65.245 | 64.262 | 47.683 | 63.28 | 58.411 |
|  | 10% | 39.67 | 17.5 | 19.304 | 18.944 | 21.327 | 15.738 | 30.366 | 17.914 | 27.57 |
|  | 20% | 7.856 | 3.25 | 16.456 | 6.832 | 7.267 | 10.82 | 12.317 | 9.804 | 10.28 |
|  | 30% | 1.552 | 3.5 | 7.595 | 1.242 | 3.633 | 3.934 | 5.732 | 4.813 | 0.467 |
|  | 40% | 1.261 | 2 | 2.215 | 0.932 | 1.896 | 1.639 | 2.683 | 1.248 | 2.336 |
|  | 50% | 0.97 | 2.5 | 0.633 | 0 | 0.632 | 2.951 | 1.098 | 2.05 | 0 |
|  | 60% | 0.097 | 0.25 | 0 | 0 | 0 | 0.328 | 0 | 0.267 | 0.467 |
|  | 70% | 0 | 0.75 | 0.316 | 0 | 0 | 0 | 0 | 0.267 | 0 |
|  | 80% | 0 | 0 | 0 | 0 | 0 | 0.328 | 0.122 | 0.089 | 0 |
|  | 90% | 0.194 | 0 | 0 | 0 | 0 | 0 | 0 | 0.267 | 0 |
|  | 100% | 0 | 0.25 | 0 | 0 | 0 | 0 | 0 | 0 | 0.467 |
| **Self-ranked household wealth status** | 1 – richest 10^th^ | 6.79 | 5.25 | 2.532 | 2.174 | 4.739 | 3.934 | 5.61 | 3.743 | 3.271 |
|  | 2 | 5.723 | 4.5 | 10.443 | 4.037 | 6.635 | 3.607 | 6.096 | 5.437 | 6.075 |
|  | 3 | 8.341 | 9.5 | 9.177 | 4.348 | 8.689 | 5.246 | 9.756 | 10.339 | 3.271 |
|  | 4 | 9.893 | 8.25 | 5.063 | 5.901 | 11.374 | 5.246 | 9.268 | 11.676 | 3.738 |
|  | 5 | 18.817 | 12.25 | 12.342 | 14.907 | 15.008 | 14.426 | 15.123 | 13.012 | 8.411 |
|  | 6 | 11.736 | 11.75 | 13.924 | 16.149 | 10.427 | 15.41 | 8.658 | 12.21 | 10.28 |
|  | 7 | 10.475 | 12 | 11.392 | 8.696 | 7.899 | 13.443 | 10.61 | 11.23 | 11.215 |
|  | 8 | 10.184 | 13.5 | 9.177 | 13.665 | 12.638 | 15.41 | 10.122 | 13.547 | 14.486 |
|  | 9 | 8.438 | 11.5 | 8.544 | 13.975 | 12.638 | 19.344 | 13.537 | 8.289 | 15.888 |
|  | 10 – poorest 10th | 9.602 | 11.5 | 17.405 | 16.149 | 9.953 | 3.934 | 11.22 | 10.517 | 23.364 |

**Table S2** The percentage of IPV acceptance amongst those holding community roles and by the respondents self- ranked household wealth and social status.

|  | Wife beating acceptance % | |
| --- | --- | --- |
|  | No | Yes |
| Community role (n) | | |
| Religious leader (39) | 74.4 | 25.6 |
| Traditional birth attendant (91) | 83.5 | 16.5 |
| Other (86) | 84.9 | 15.1 |
| Militia (63) | 88.9 | 11.1 |
| None (4769) | 90.7 | 9.3 |
| Teacher (99) | 91.9 | 8.1 |
| Kebele leader (12) | 100 | 0 |
| Self-ranked household wealth and status | | |
| 1 – richest 10^th^ (243) | 85.6 | 14.4 |
| 2 (300) | 82 | 18 |
| 3 (441) | 85.3 | 14.7 |
| 4 (473) | 85.4 | 15.6 |
| 5 (757) | 87.2 | 12.8 |
| 6 (607) | 92.1 | 7.9 |
| 7 (548) | 92.3 | 7.7 |
| 8 (625) | 93.9 | 6.1 |
| 9 (582) | 94.3 | 5.7 |
| 10 – poorest 10^th^ (587) | 96.9 | 3.1 |

**Table S3** Centrality measures for the chatting and respect networks dependent on IPV acceptance and community role.

| **Network** | **Statistic** | **IPV**  **accepting** | **IPV non-**  **accepting** | **No role** | **Religious**  **leader** | **Kebele**  **leader** | **Traditional**  **birth**  **attendant** | **Teacher** | **Militia** | **Other** |
| --- | --- | --- | --- | --- | --- | --- | --- | --- | --- | --- |
| ***In-degree*** | | | | | | | | | | |
| **Chatting** | **Median** | 1 | 1 | 1 | 2 | 3 | 1 | 1 | 2 | 2 |
|  | **Range** | 0-9 | 0-11 | 0-11 | 0-10 | 0-10 | 0-5 | 0-9 | 0-6 | 0-8 |
| **Respect** | **Median** | 0 | 0 | 0 | 2 | 4.5 | 0 | 0 | 1 | 1 |
|  | **Range** | 0-44 | 0-176 | 0-117 | 0-176 | 0-21 | 0-11 | 0-41 | 0-22 | 0-43 |
| ***Out-degree*** | | | | | | | | | | |
| **Chatting** | **Median** | 0 | 0 | 0 | 2 | 3 | 0 | 0 | 2 | 2 |
|  | **Range** | 0-6 | 0-9 | 0-9 | 0-5 | 0-5 | 0-5 | 0-5 | 0-5 | 0-7 |
| **Respect** | **Median** | 0 | 0 | 0 | 2 | 2 | 0 | 1 | 2 | 2 |
|  | **Range** | 0-8 | 0-8 | 0-8 | 0-6 | 0-5 | 0-5 | 0-4 | 0-5 | 0-5 |
| ***Vertex betweeness*** | | | | | | | | | | |
| **Chatting** | **Median** | 0 | 0 | 0 | 0 | 87.125 | 0 | 0 | 0 | 0 |
|  | **Range** | 0-34,362 | 0-38,219 | 0- 38,219 | 0-20,054 | 0-34,739 | 0-34,189 | 0-33,624 | 0-36,425 | 0-31,901 |
| **Respect** | **Median** | 0 | 0 | 0 | 0 | 4 | 0 | 0 | 0 | 0 |
|  | **Range** | 0- 8093 | 0- 16,957 | 0-11,991 | 0-4488 | 0-16,957 | 0-10,022 | 0-8126 | 0-5973 | 0-8093 |
| ***Harmonic centrality*** | | | | | | | | | | |
| **Chatting** | **Median** | 0 | 0 | 0 | 5.917 | 9.958 | 0 | 0 | 3 | 3.25 |
|  | **Range** | 0-94.497 | 0-92.633 | 0-94.497 | 0-60.024 | 0-73.943 | 0-84.523 | 0-92.633 | 0-76.163 | 0-68.874 |
| **Respect** | **Median** | 0 | 0 | 0 | 4.333 | 7.161 | 0 | 1 | 4.167 | 3 |
|  | **Range** | 0-25.129 | 0-28.238 | 0-28.238 | 0-25.106 | 0-23.475 | 0-23.357 | 0-21.935 | 0-23.689 | 0-26.57 |

**Table S4** The percentage distribution of alter IPVAW acceptance dependent on egos acceptance within the household co-residency network. Abbreviations: head of household (HHD).

|  | | | Ego | | | | | | | |
| --- | --- | --- | --- | --- | --- | --- | --- | --- | --- | --- |
|  |  |  | Male HHD | | Wife of HHD | | Son of HHD | | Daughter of HHD | |
|  |  |  | Yes | No | Yes | No | Yes | No | Yes | No |
| Alter | Male HHD | Yes | - | - | 6.3 | 8.7 | 16.7 | 7 | 0 | 7.3 |
|  |  | No | - | - | 93.7 | 91.3 | 83.3 | 93 | 100 | 92.7 |
|  | Wife of HHD or female HHD | Yes | 11.3 | 17.6 | - | - | 7.7 | 5.1 | 39.1 | 9.3 |
|  |  | No | 88.7 | 82.4 | - | - | 92.3 | 94.9 | 60.9 | 90.7 |

**Table S5** The percentage distribution of same-gender ties dependent on ego and alter gender and IPVAW acceptance in the chatting and respect networks.

|  | | Man | | Woman | |
| --- | --- | --- | --- | --- | --- |
|  |  | Yes | No | Yes | No |
| Chatting | | | | | |
| Man | Yes | 1.8 | 2.8 | - | - |
|  | No | 2.2 | 47.1 | - | - |
| Woman | Yes | - | - | 3.5 | 2.3 |
|  | No | - | - | 2.1 | 38.2 |
| Respect | | | | | |
| Man | Yes | 2.1 | 4.2 | - | - |
|  | No | 2.7 | 57.4 | - | - |
| Women | Yes | - | - | 3 | 1.6 |
|  | No | - | - | 0.8 | 28.1 |

**Table S6** The percentage distribution of mixed-gender ties dependent on ego-alter gender combination and IPVAW acceptance in the chatting and respect networks.

|  | | Man | | Woman | |
| --- | --- | --- | --- | --- | --- |
|  |  | Yes | No | Yes | No |
| Chatting | | | | | |
| Man | Yes | - | - | 0.3 | 5.8 |
|  | No | - | - | 9.4 | 51.2 |
| Woman | Yes | 0.3 | 2.7 | - | - |
|  | No | 2.1 | 28.3 | - | - |
| Respect | | | | | |
| Man | Yes | - | - | 0.9 | 5.8 |
|  | No | - | - | 11 | 71 |
| Women | Yes | 0.1 | 0.8 | - | - |
|  | No | 1.2 | 9.2 | - | - |

**Table S7** Structural characteristics at the network-level of the subsample who reported their networks (n = 2545). For the interpretation of density, reciprocity and transitivity see the ‘Materials and Methods’ section. The number of isolates reflects the number of individuals who were either not named in response to the name generator or reported an entirely unidentified network.

| Characteristic | Chatting | Respect |
| --- | --- | --- |
| Network type | Directed | Directed |
| Ties (n) | 4304 | 4209 |
| Mean out-degree | 1.691 | 1.654 |
| Range out-degree | 0-6 | 0-6 |
| Mean in-degree | 1.691 | 1.654 |
| Range in-degree | 0-11 | 0-117 |
| Density | 0.001 | 0.001 |
| Reciprocity | 0.317 | 0.046 |
| Transitivity | 0.232 | 0.079 |
| Isolates | 177 | 253 |

**Figure S1** Violin plot of the distribution of the ages of male head’s of household (n = 1601) dependent on their self-ranked household wealth and status. The point estimate reflects the mean and the error bar is the standard deviation.

**
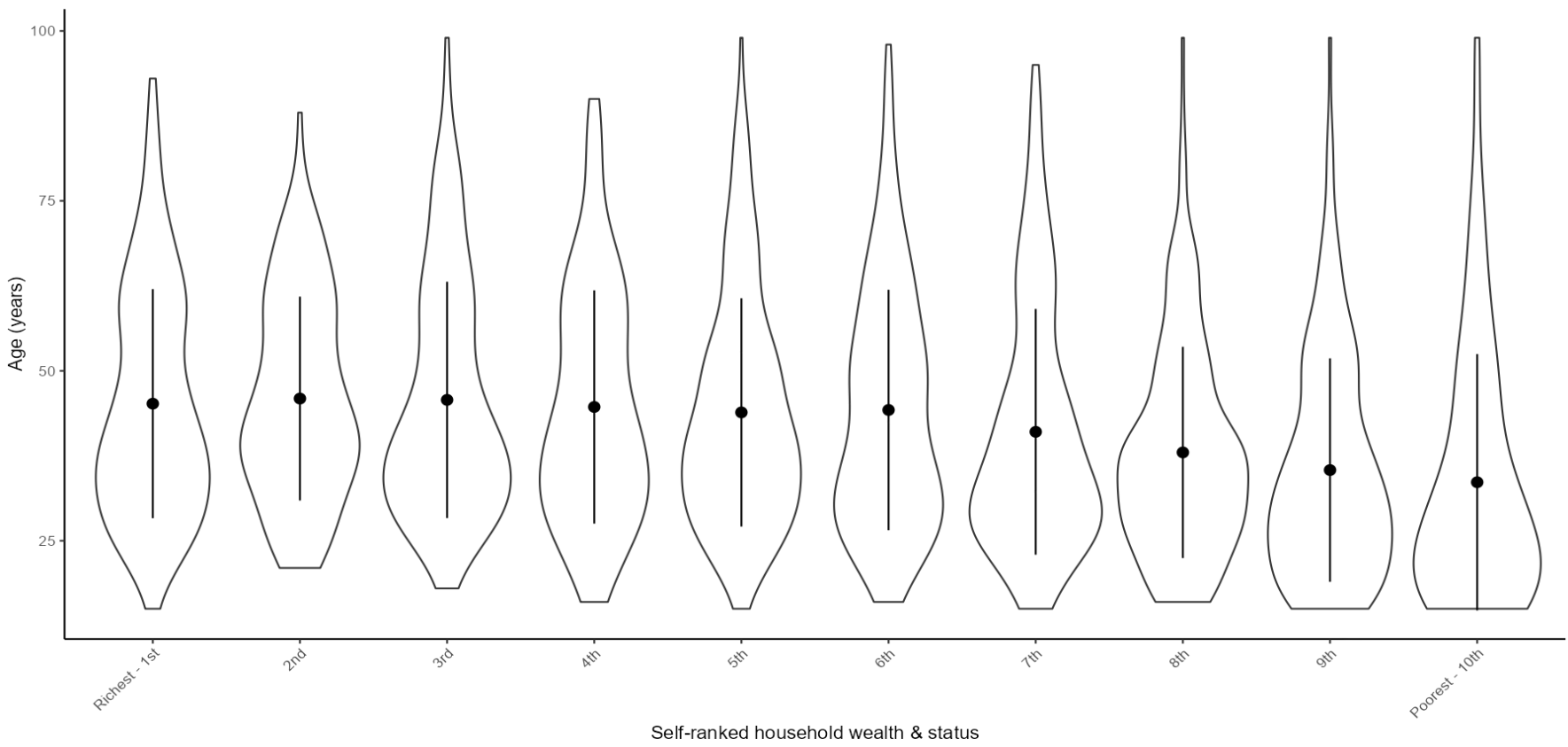
**

**Figure S2** Plot of the percentage distribution of people (n = 5163) self-ranking their household in either the poorest or richest half of their village dependent on the ranker’s highest level of education.

**
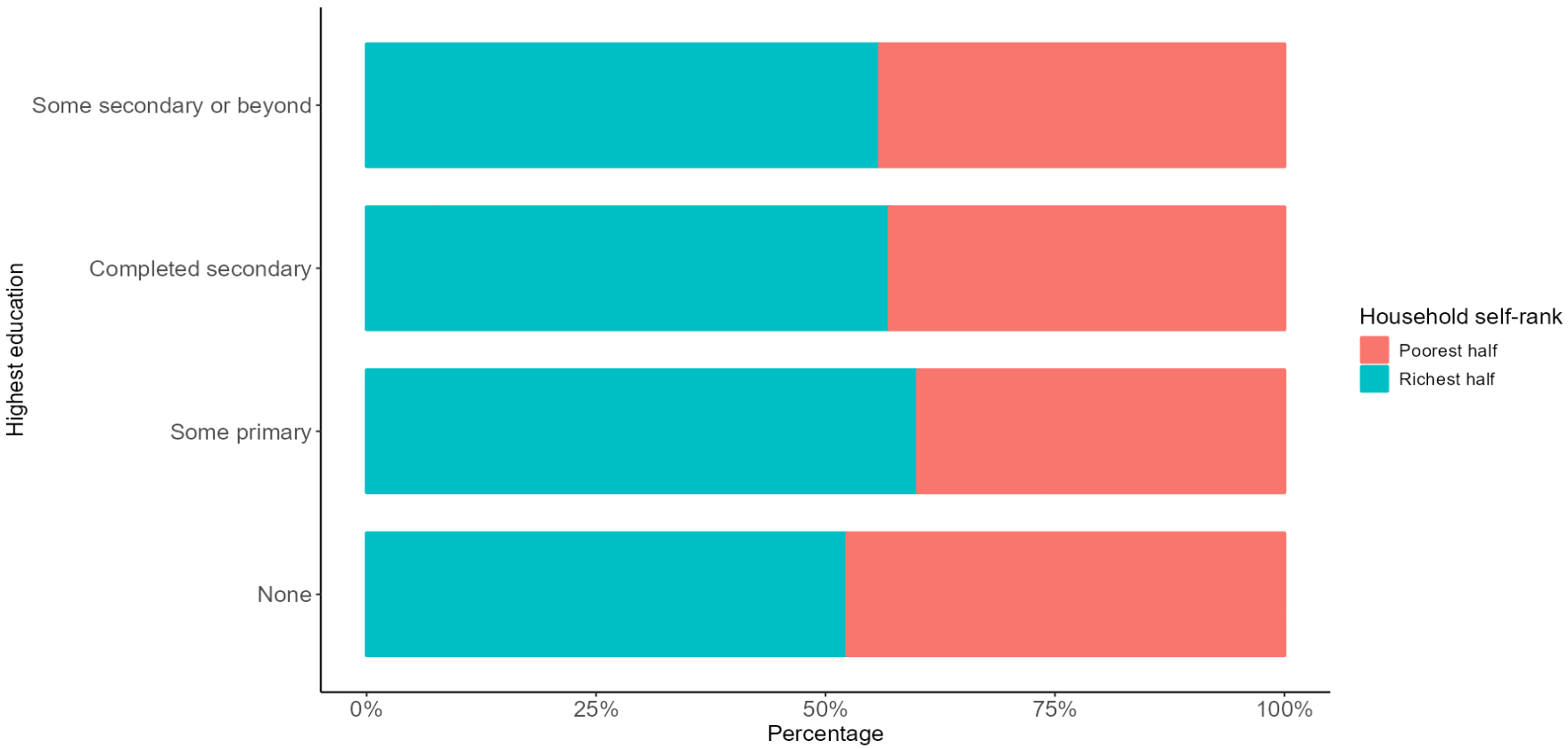
**

**Figure S3** Violin plot of the distribution of the ages of people (n = 5163) dependent on their highest level of education. The point estimate reflects the mean and the error bar is the standard deviation.

**
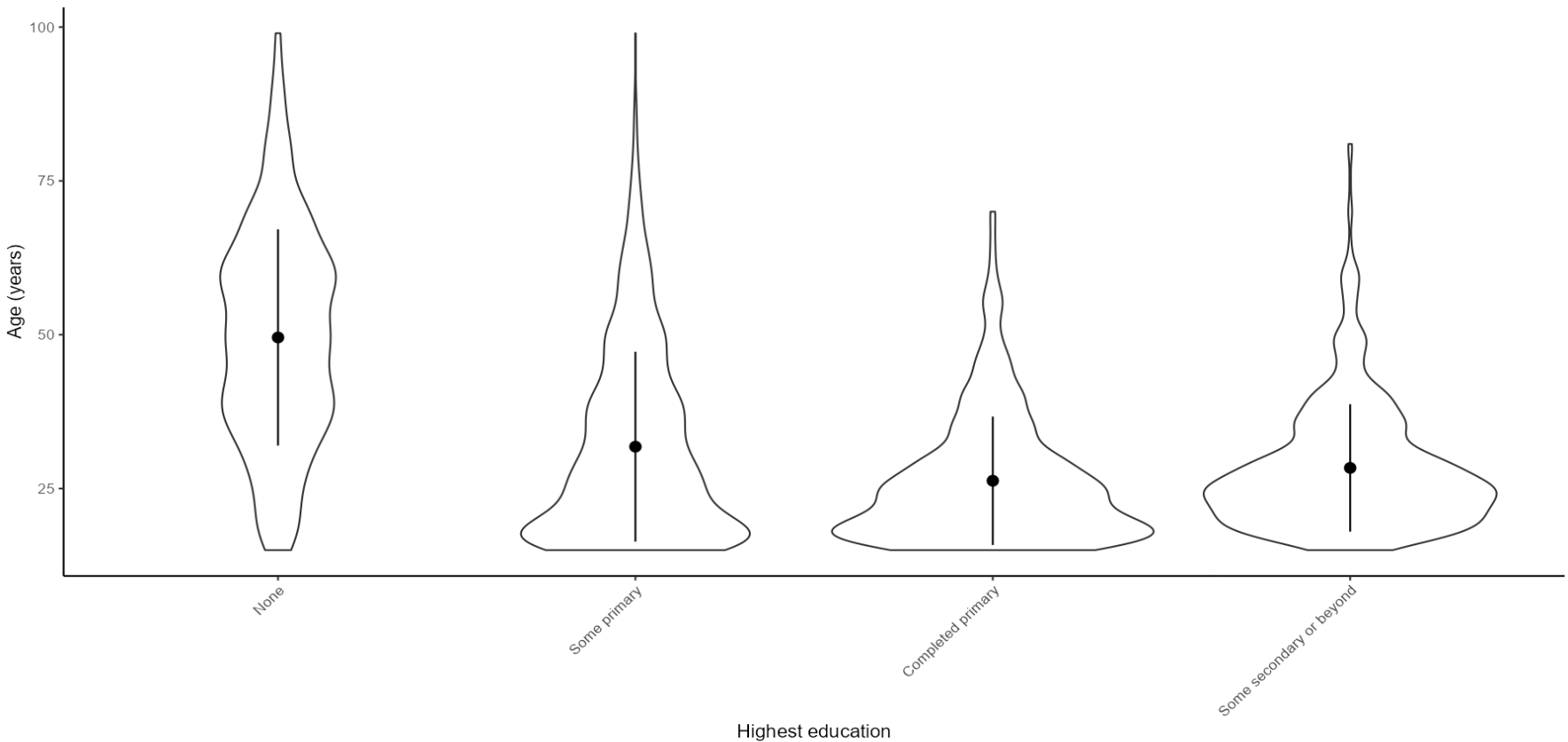
**

**Figure S4** Graph of the respect network. Nodes (i.e. people, n = 5163) are coloured by IPVAW (non)acceptance and shaped by whether or not they were asked to report their network ties.


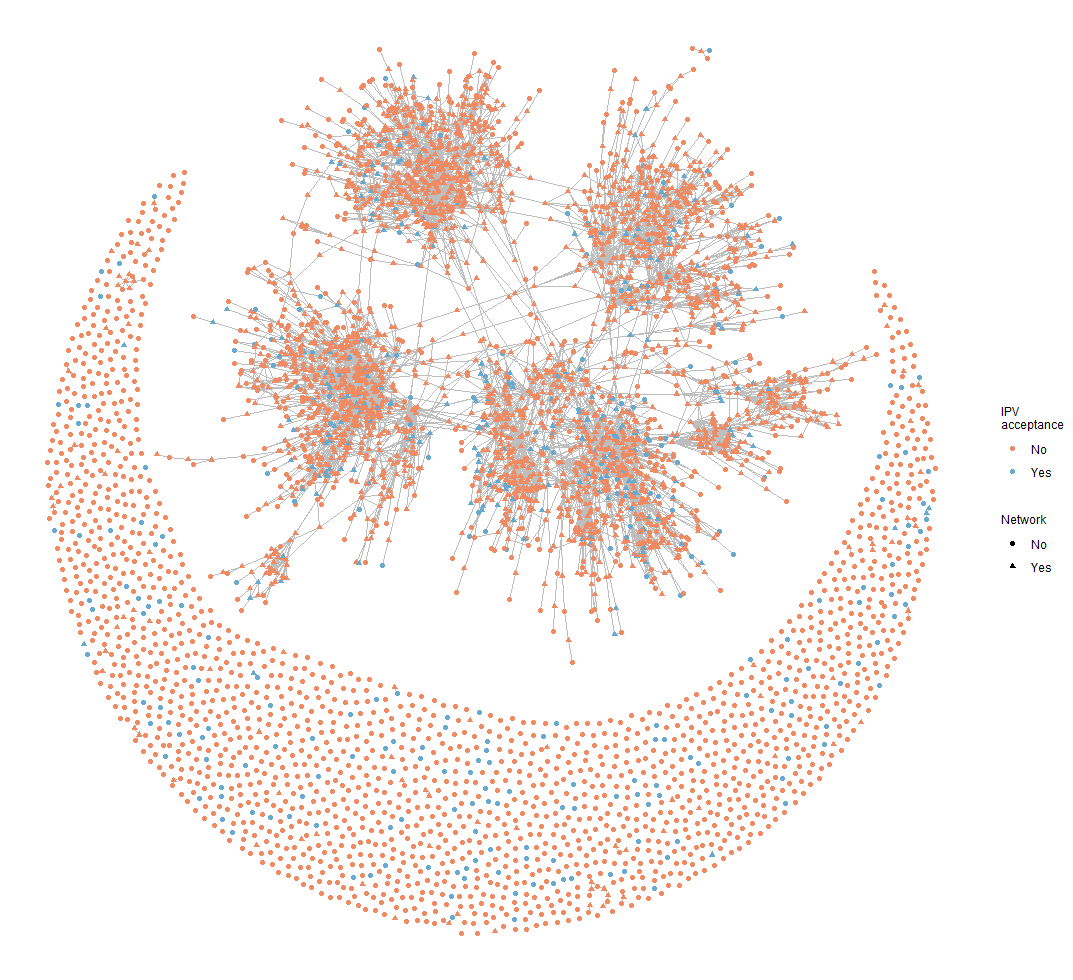


**Figure S5** Graph of the chatting network of within-village ties in village 1. Nodes (i.e. people, n = 1031) are coloured by IPVAW (non)acceptance and shaped by gender.


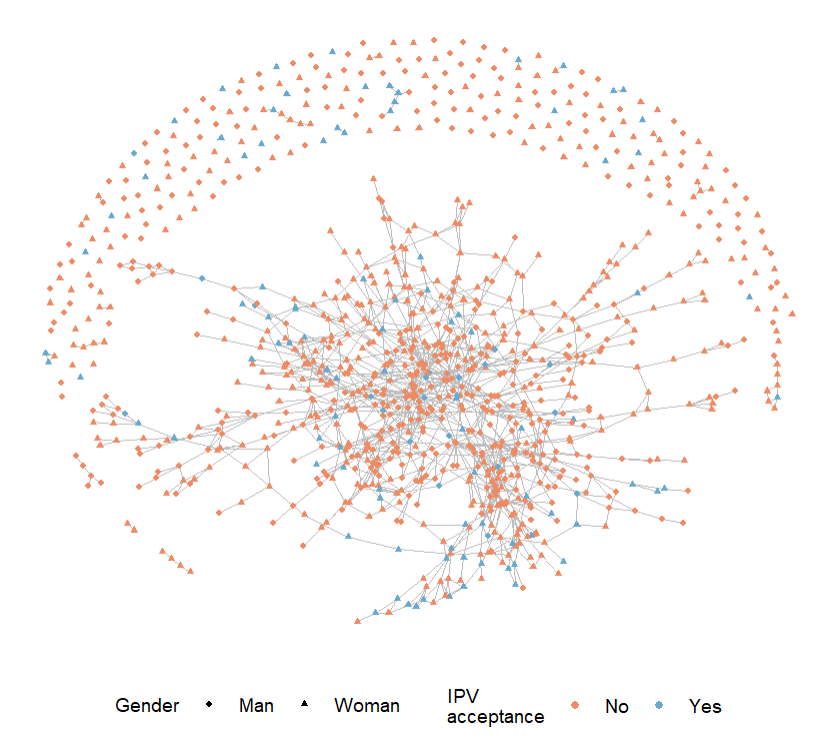


**Figure S6** Graph of the chatting network of within-village ties in village 2. Nodes (i.e. people, n = 400) are coloured by IPVAW (non)acceptance and shaped by gender.


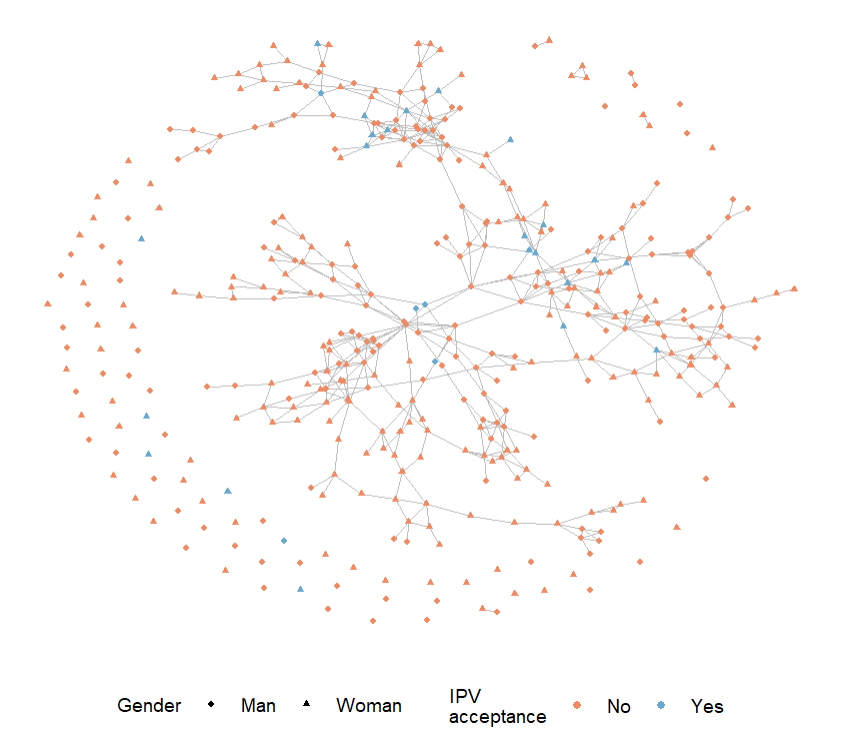


**Figure S7** Graph of the chatting network of within-village ties in village 3. Nodes (i.e. people, n = 316) are coloured by IPVAW (non)acceptance and shaped by gender.


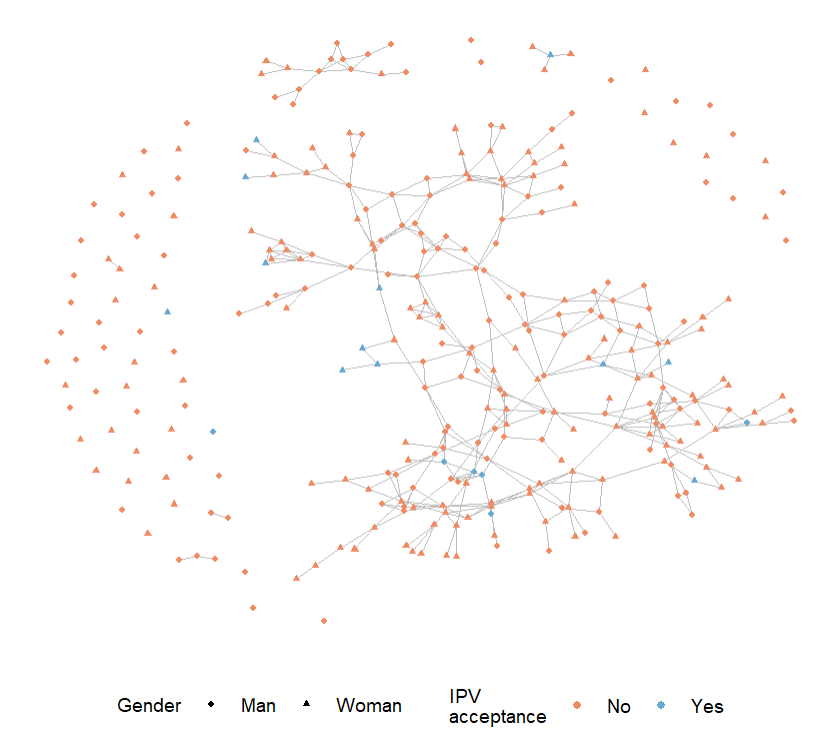


**Figure S8** Graph of the chatting network of within-village ties in village 5. Nodes (i.e. people, n = 633) are coloured by IPVAW (non)acceptance and shaped by gender.


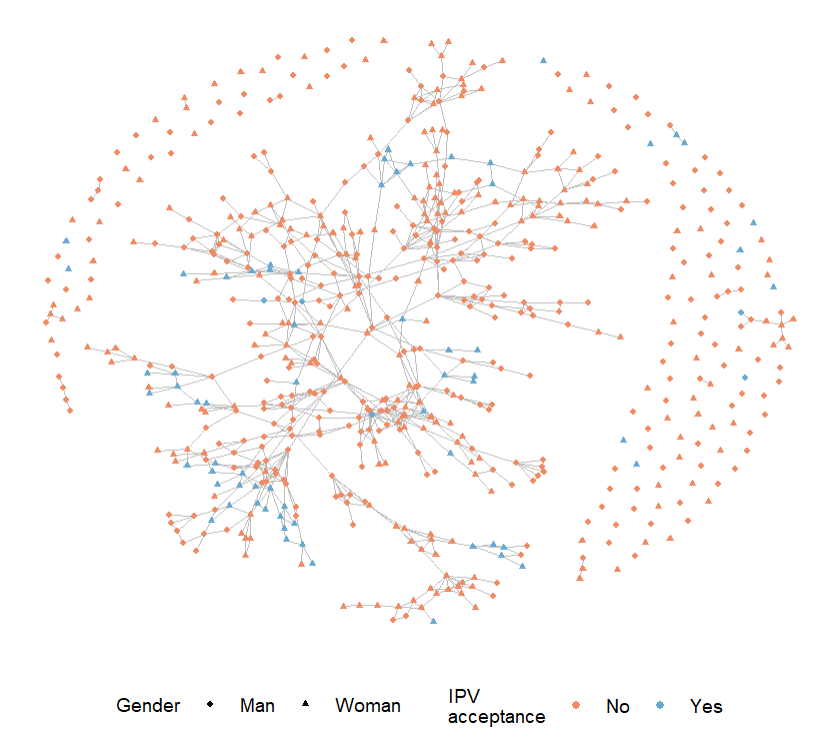


**Figure S9** Graph of the chatting network of within-village ties in village 6. Nodes (i.e. people, n = 305) are coloured by IPVAW (non)acceptance and shaped by gender.


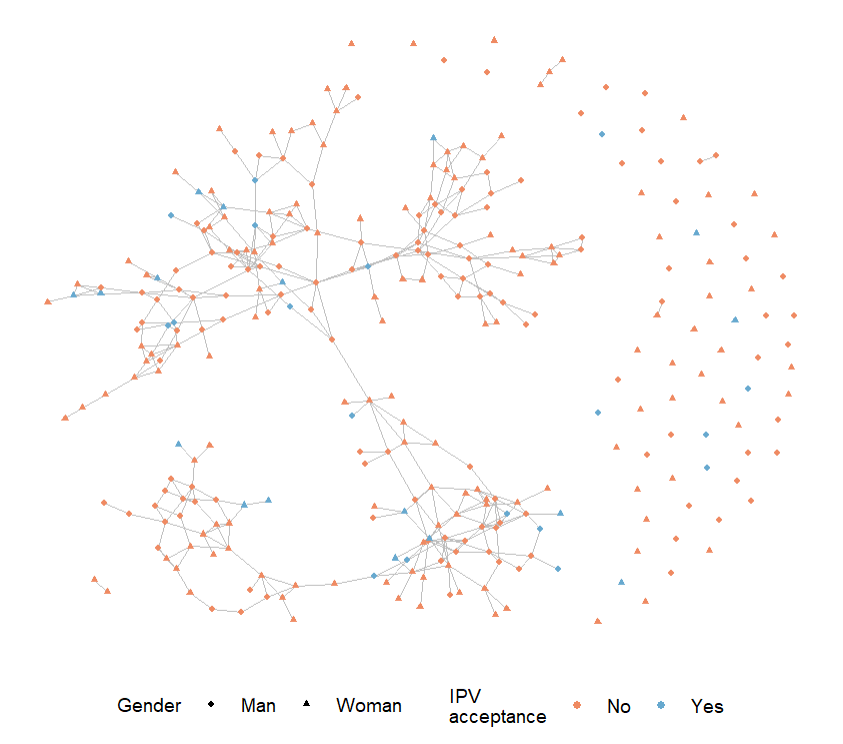


**Figure S10** Graph of the chatting network of within-village ties in village 8. Nodes (i.e. people, n = 1122) are coloured by IPVAW (non)acceptance and shaped by gender.


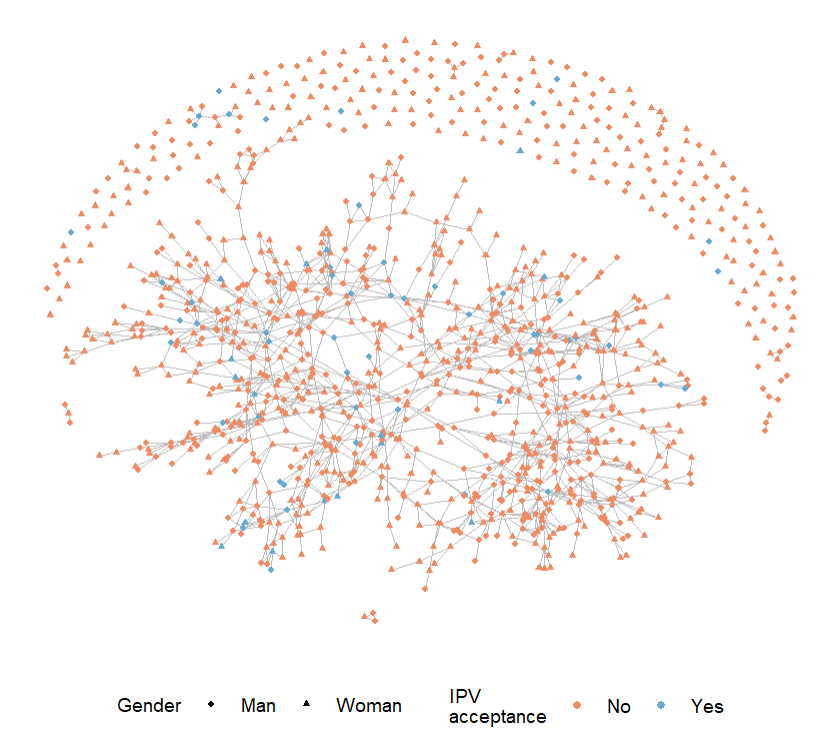


**Figure S11** Graph of the chatting network of within-village ties in village 9. Nodes (i.e. people, n = 214) are coloured by IPVAW (non)acceptance and shaped by gender.

**
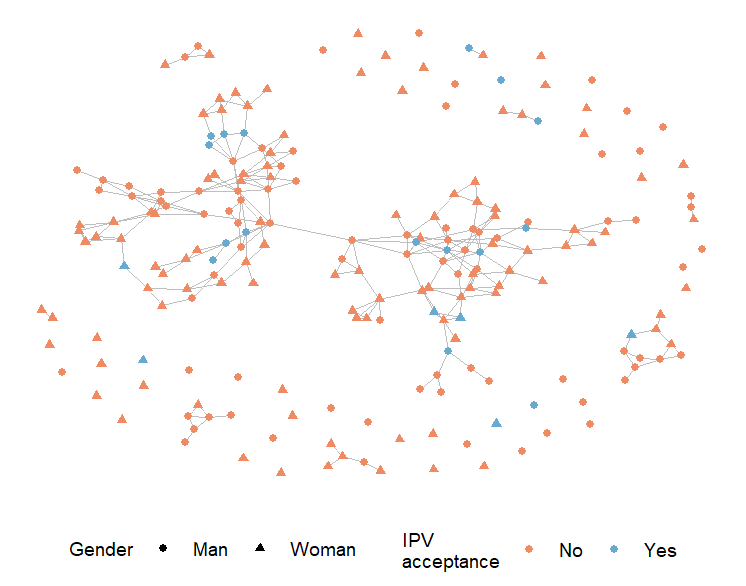
**

**Figure S12** Graph of the respect network of within-village ties in village 1. Nodes (i.e. people, n = 1031) are coloured by IPVAW (non)acceptance and shaped by gender.


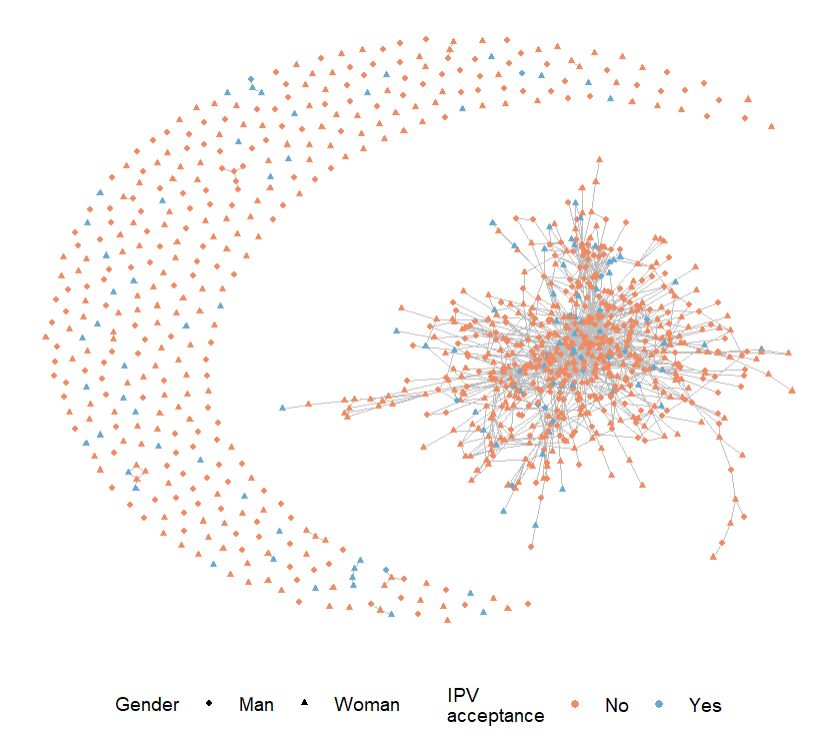


**Figure S13** Graph of the respect network of within-village ties in village 2. Nodes (i.e. people, n = 400) are coloured by IPVAW (non)acceptance and shaped by gender.


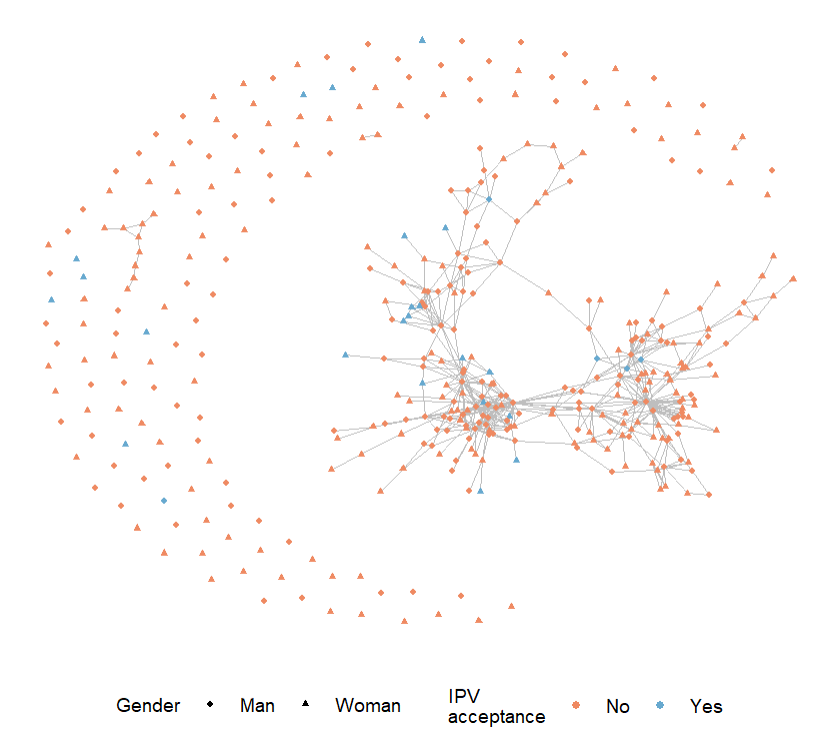


**Figure S14** Graph of the respect network of within-village ties in village 3. Nodes (i.e. people, n = 316) are coloured by IPVAW (non)acceptance and shaped by gender.


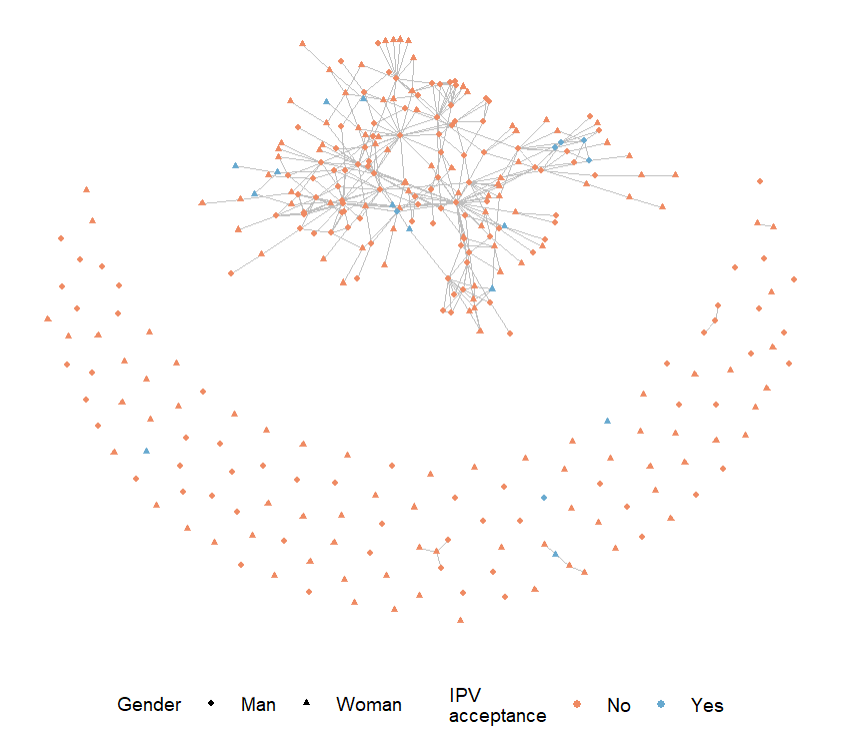


**Figure S15** Graph of the respect network of within-village ties in village 5. Nodes (i.e. people, n = 633) are coloured by IPVAW (non)acceptance and shaped by gender.


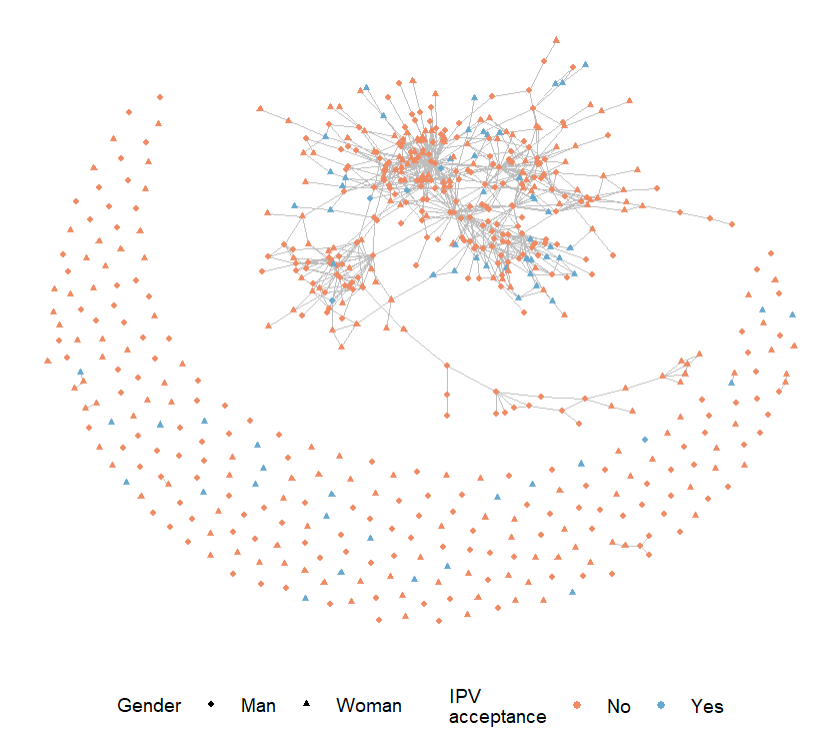


**Figure S16** Graph of the respect network of within-village ties in village 6. Nodes (i.e. people, n = 305) are coloured by IPVAW (non)acceptance and shaped by gender.


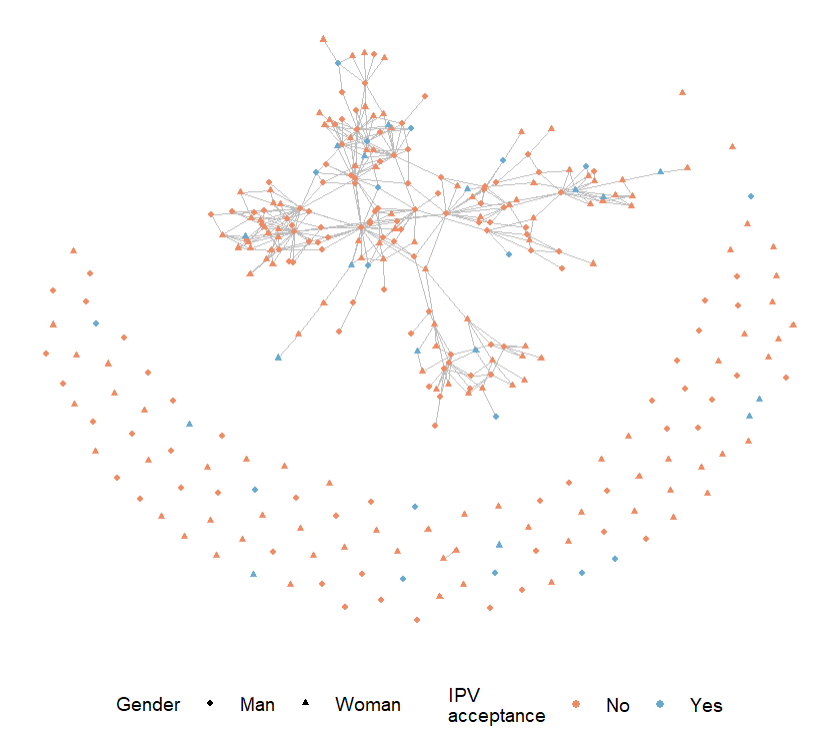


**Figure S17** Graph of the respect network of within-village ties in village 8. Nodes (i.e. people, n = 1122) are coloured by IPVAW (non)acceptance and shaped by gender.


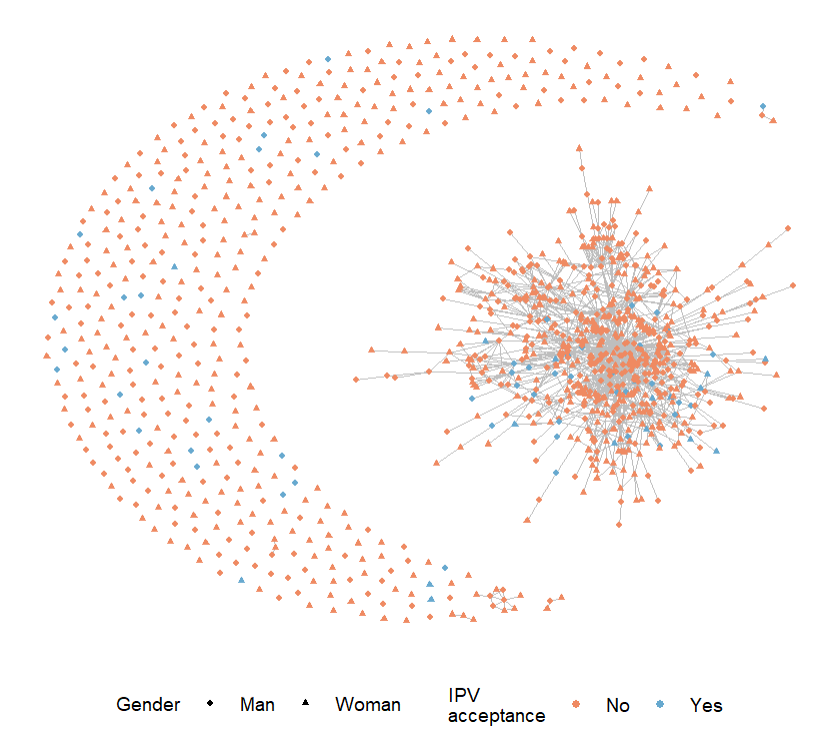


**Figure S18** Graph of the respect network of within-village ties in village 9. Nodes (i.e. people, n = 214) are coloured by IPVAW (non)acceptance and shaped by gender.


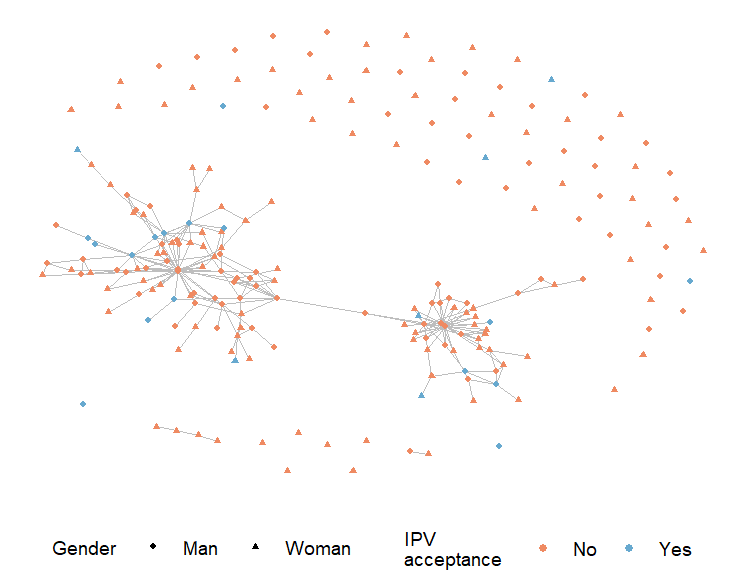


**Figure S19** Graph of the household members network in village 1. Nodes (i.e. people, n = 1031) are coloured by IPVAW (non)acceptance and shaped by gender.


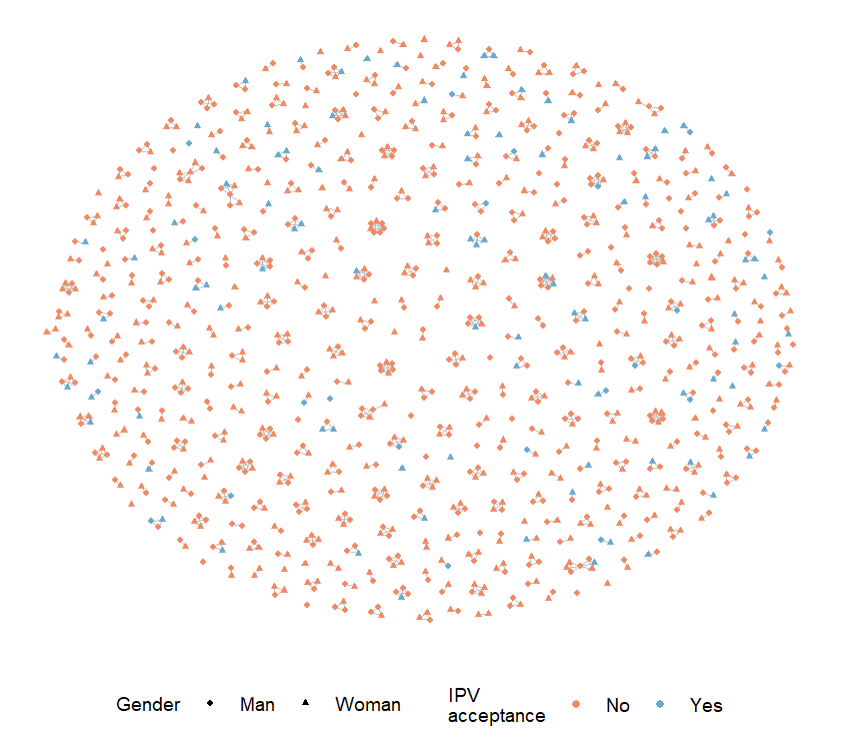


**Figure S20** Graph of the household members network in village 2. Nodes (i.e. people, n = 400) are coloured by IPVAW (non)acceptance and shaped by gender.


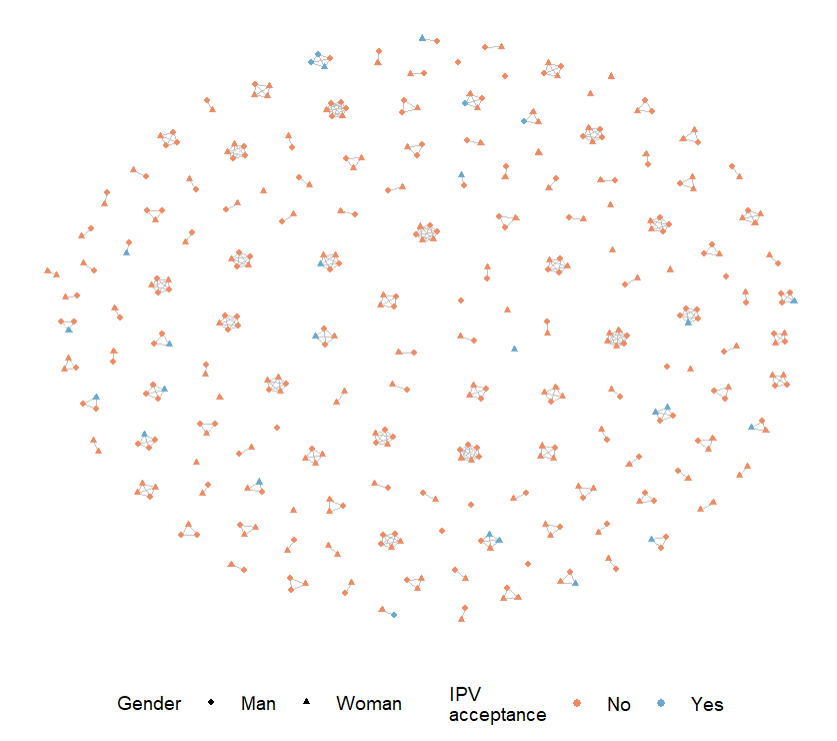


**Figure S21** Graph of the household members network in village 3. Nodes (i.e. people, n = 316) are coloured by IPVAW (non)acceptance and shaped by gender.


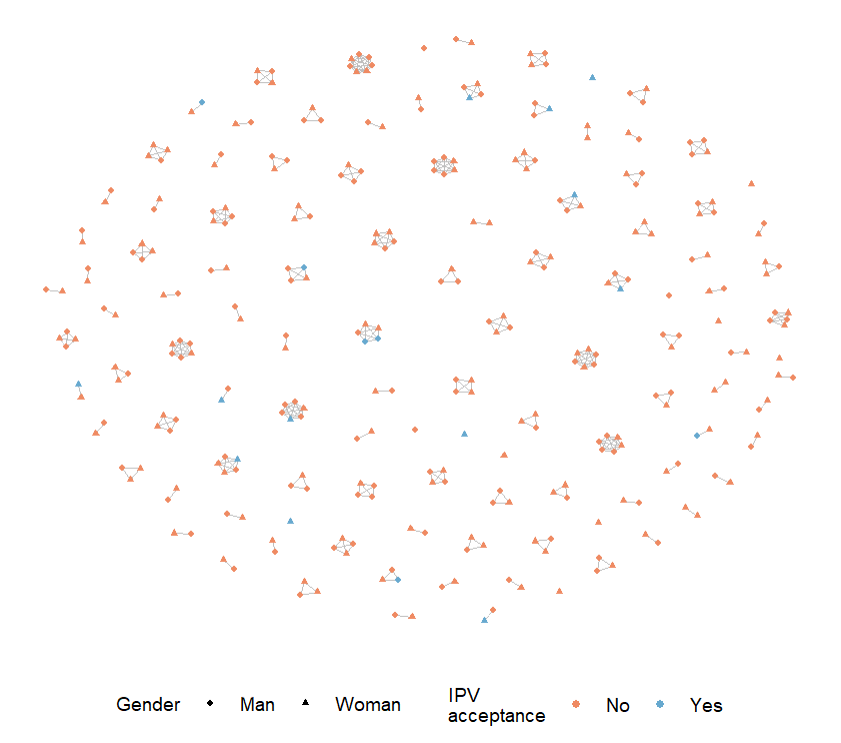


**Figure S22** Graph of the household members network in village 5. Nodes (i.e. people, n = 633) are coloured by IPVAW (non)acceptance and shaped by gender.


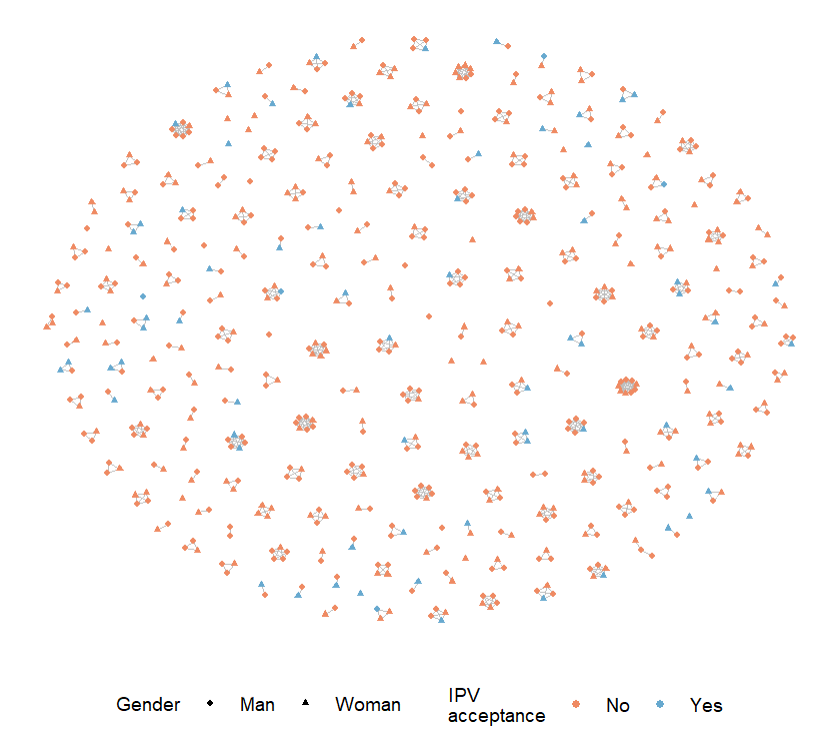


**Figure S23** Graph of the household members network in village 6. Nodes (i.e. people, n = 305) are coloured by IPVAW (non)acceptance and shaped by gender.


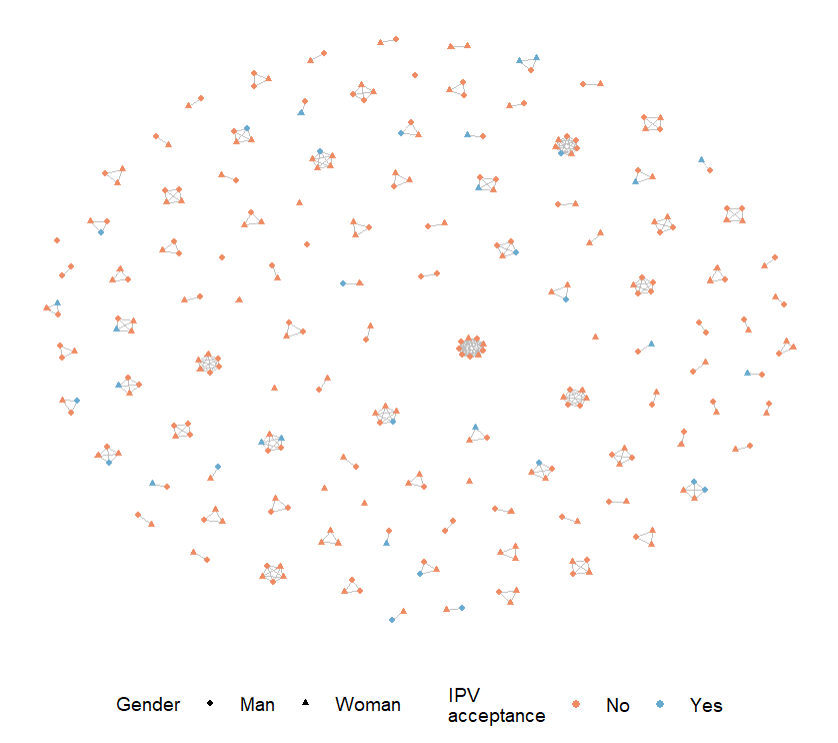


**Figure S24** Graph of the household members network in village 8. Nodes (i.e. people, n = 1122) are coloured by IPVAW (non)acceptance and shaped by gender.


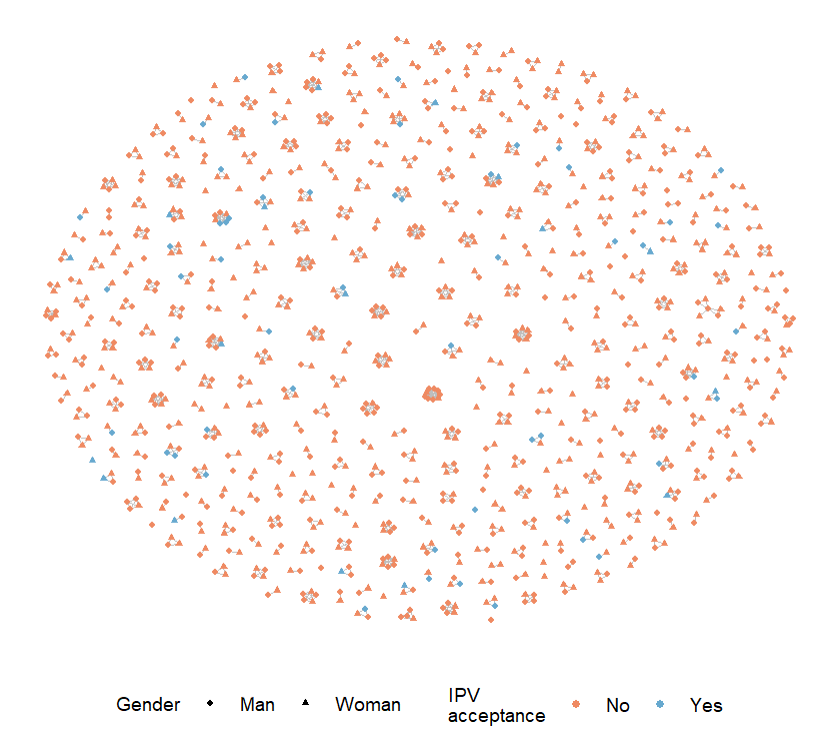


**Figure S25** Graph of the household members network in village 9. Nodes (i.e. people, n = 214) are coloured by IPVAW (non)acceptance and shaped by gender.


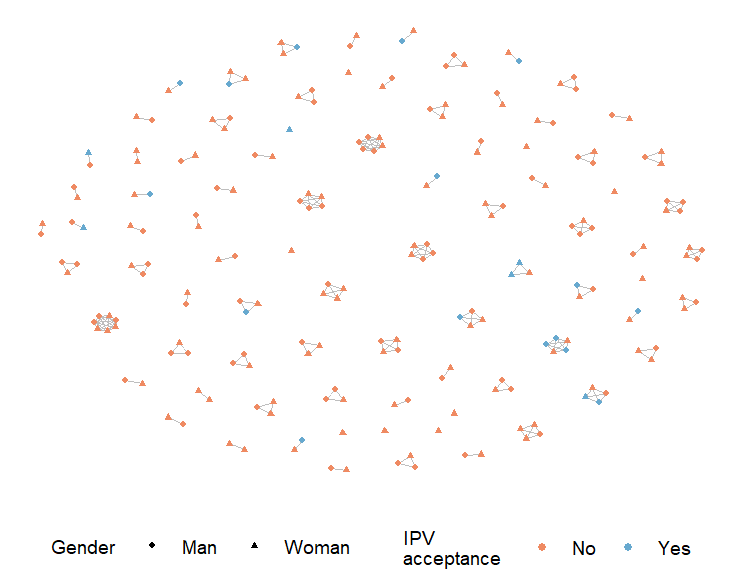


**Figure S26** Kin status of network ties nominated by IPV (non)acceptance.


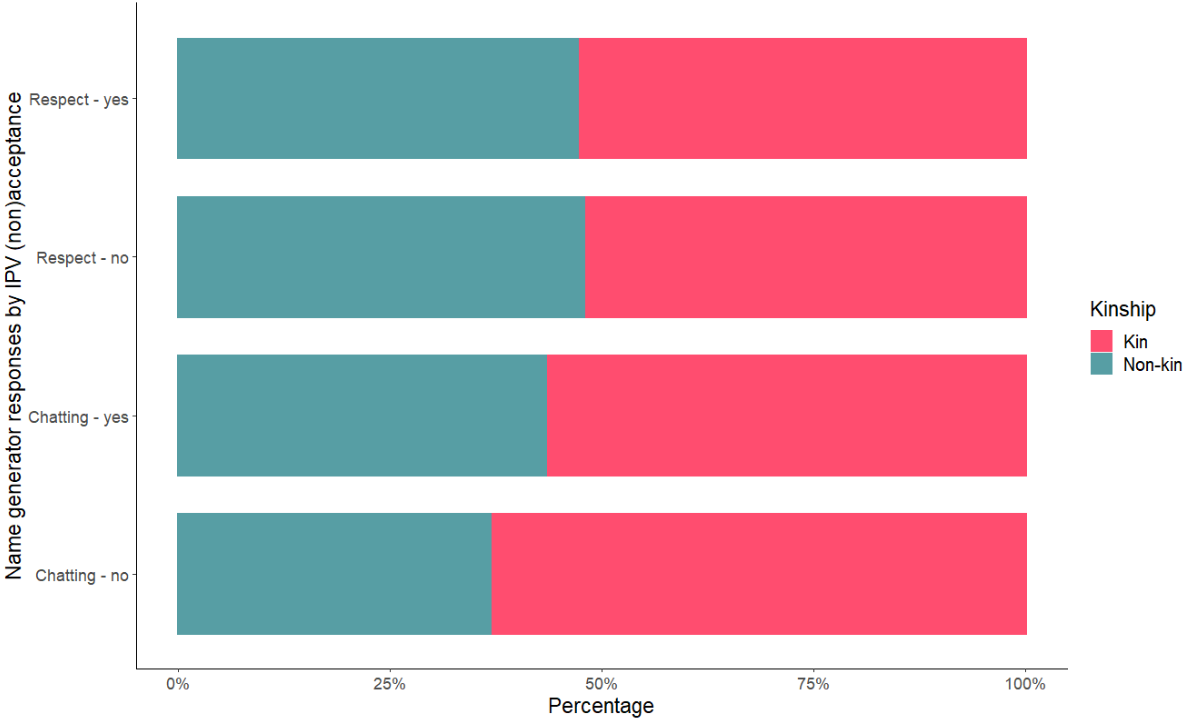


**Figure S27** Chatting nominations of men by men, split by relationship to the nominator and the nominator’s IPV acceptance


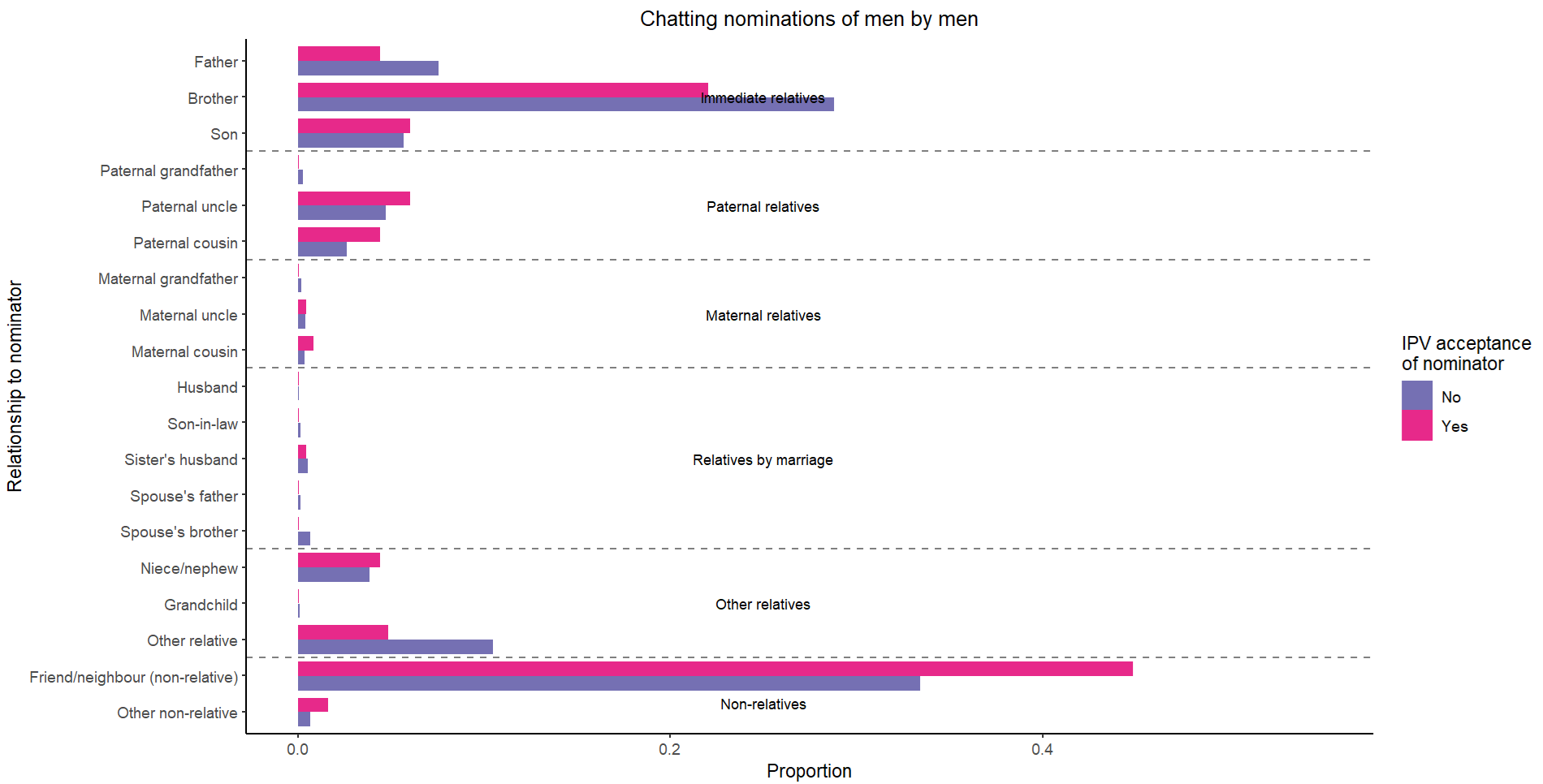


**Figure S28** Chatting nominations of women by men, split by relationship to the nominator and the nominator’s IPV acceptance


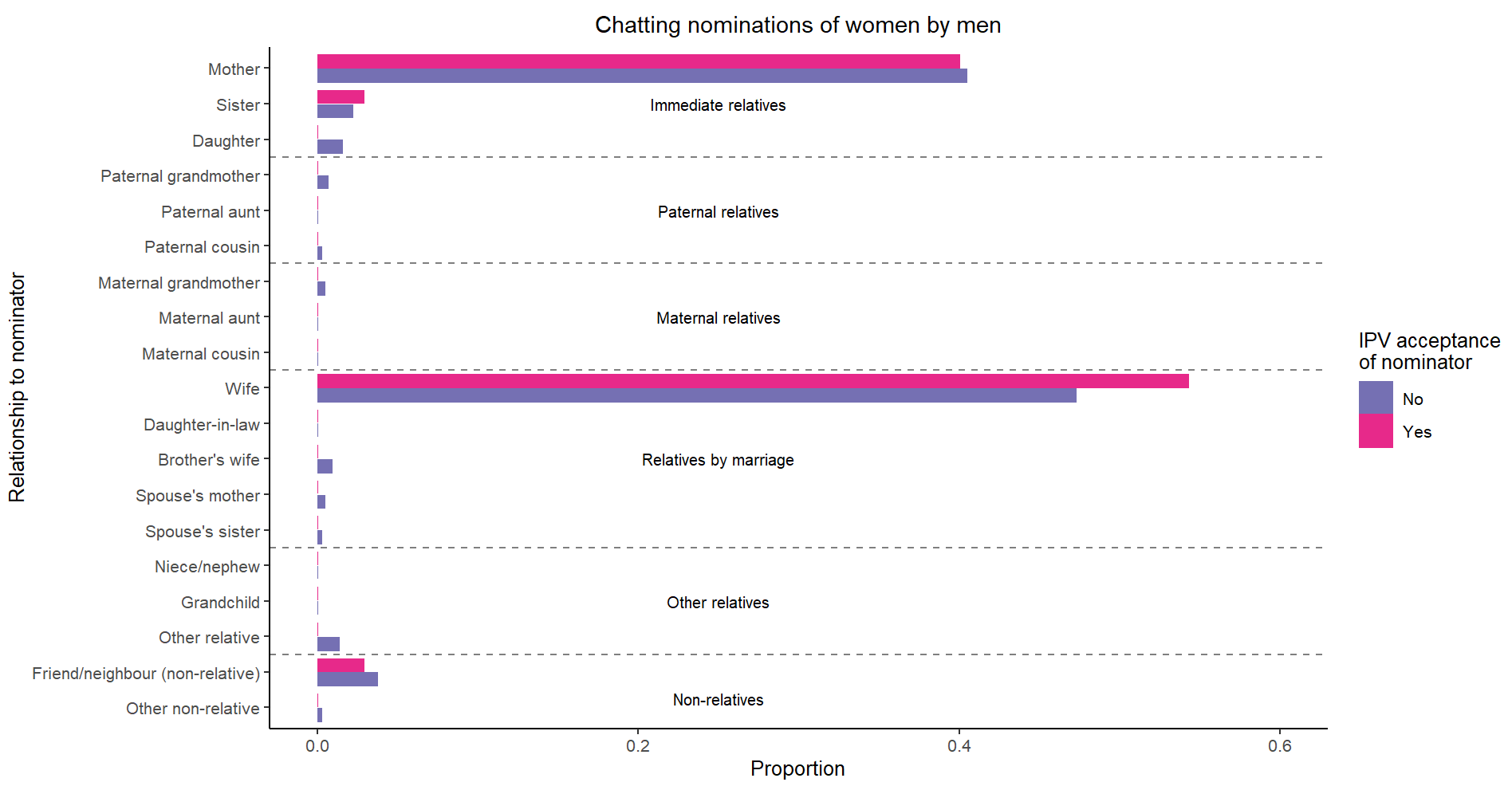


**Figure S29** Chatting nominations of women by women, split by relationship to the nominator and the nominator’s IPV acceptance


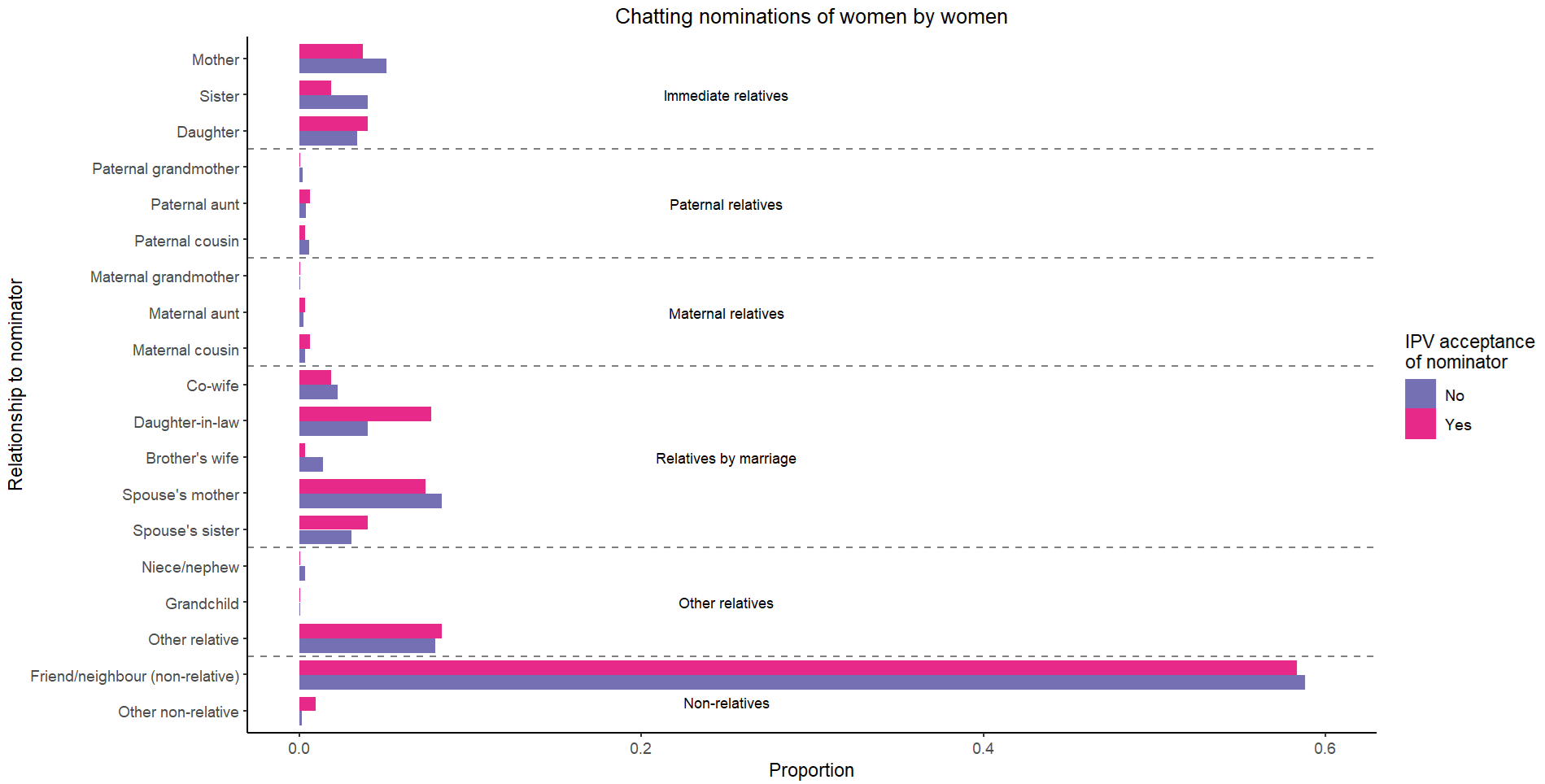


**Figure S30** Chatting nominations of men by women, split by relationship to the nominator and the nominator’s IPV acceptance


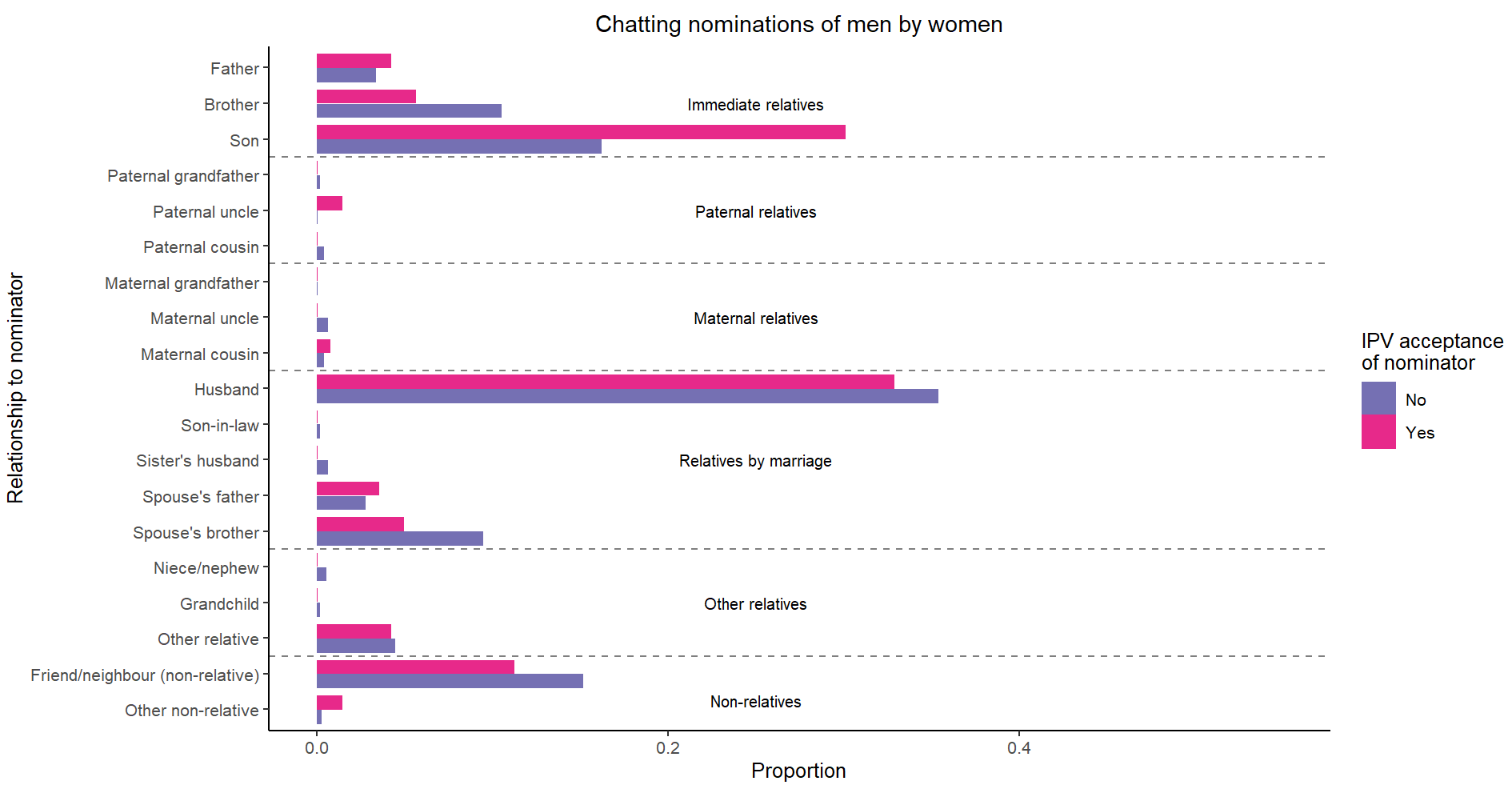


**Figure S31** Respect nominations of men by men, split by relationship to the nominator and the nominator’s IPV acceptance


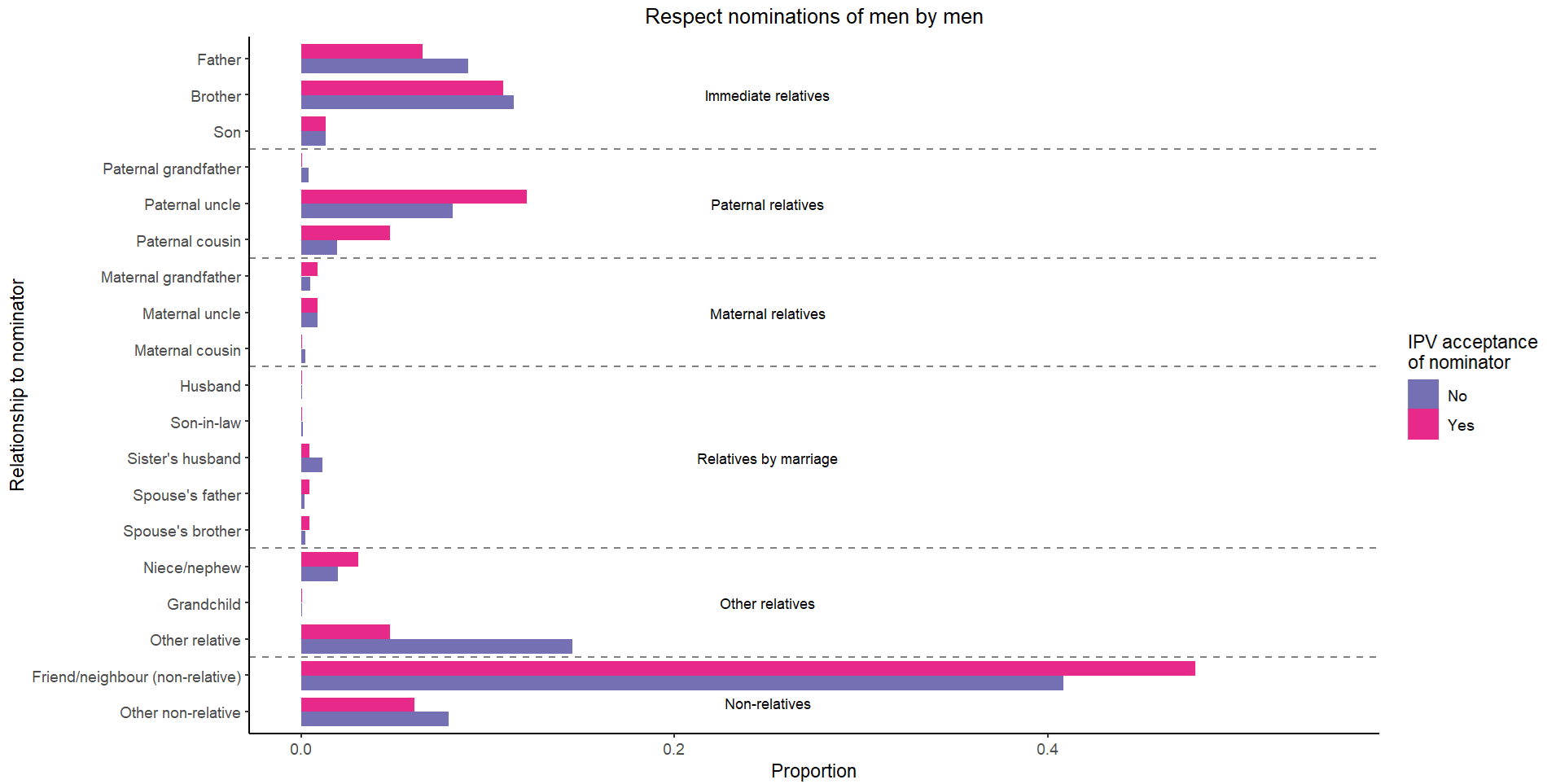


**Figure S32** Respect nominations of women by men, split by relationship to the nominator and the nominator’s IPV acceptance


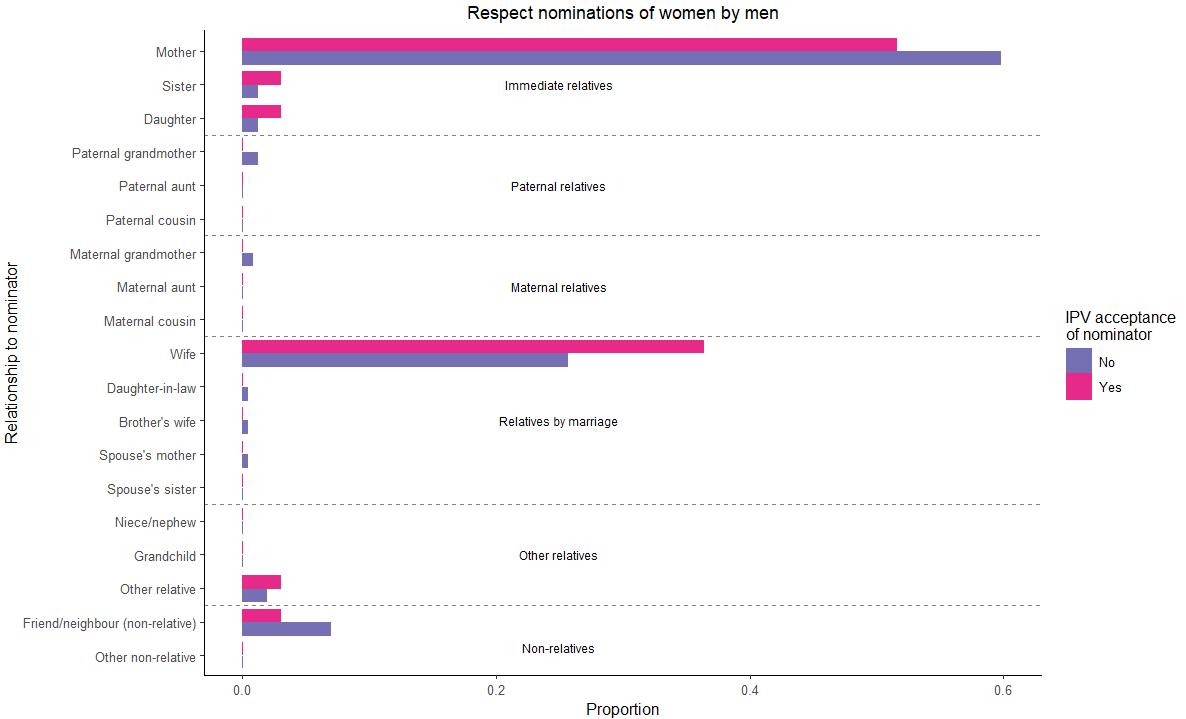


**Figure S33** Respect nominations of women by women, split by relationship to the nominator and the nominator’s IPV acceptance


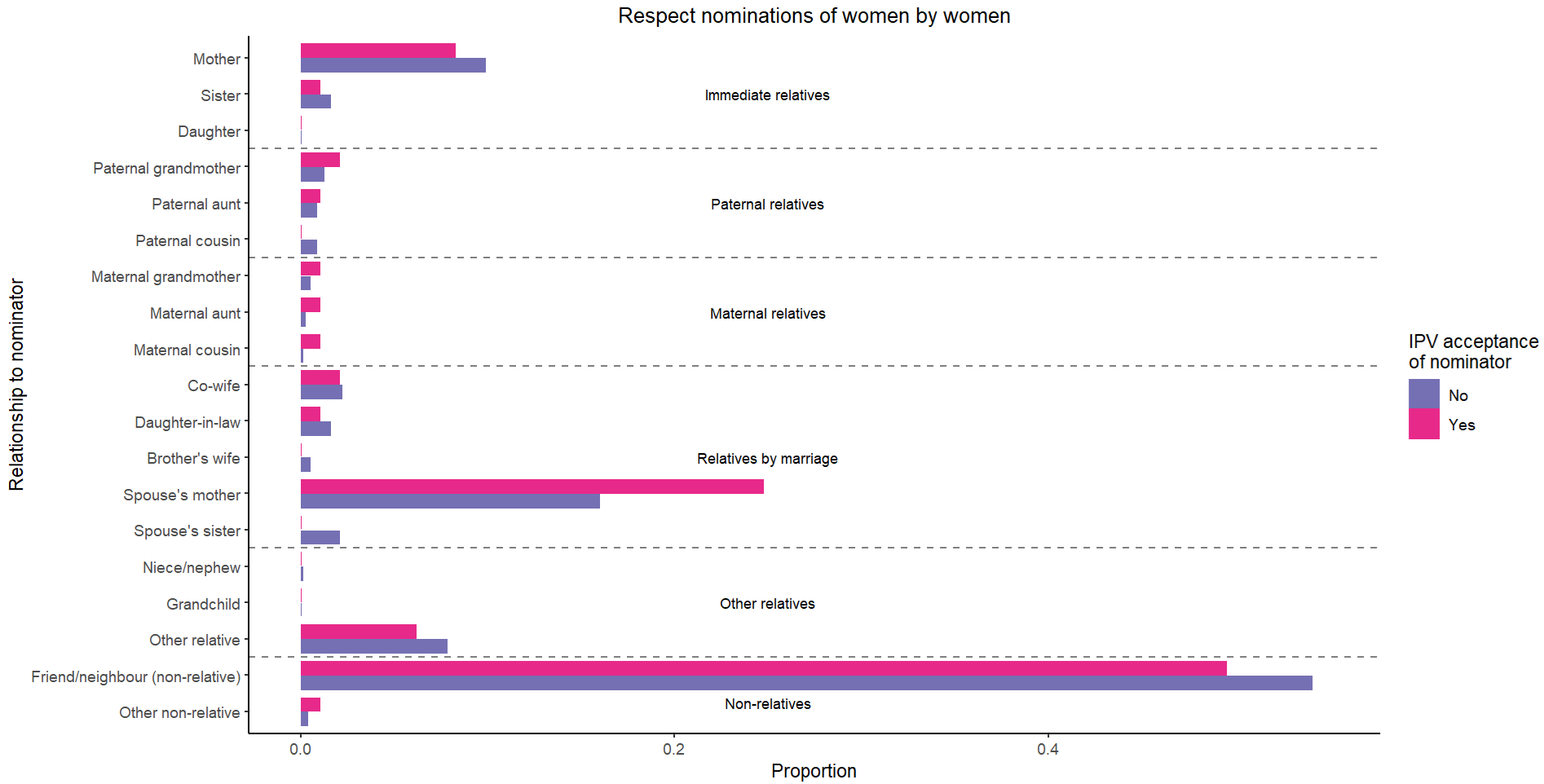


**Figure S34** Respect nominations of men by women, split by relationship to the nominator and the nominator’s IPV acceptance


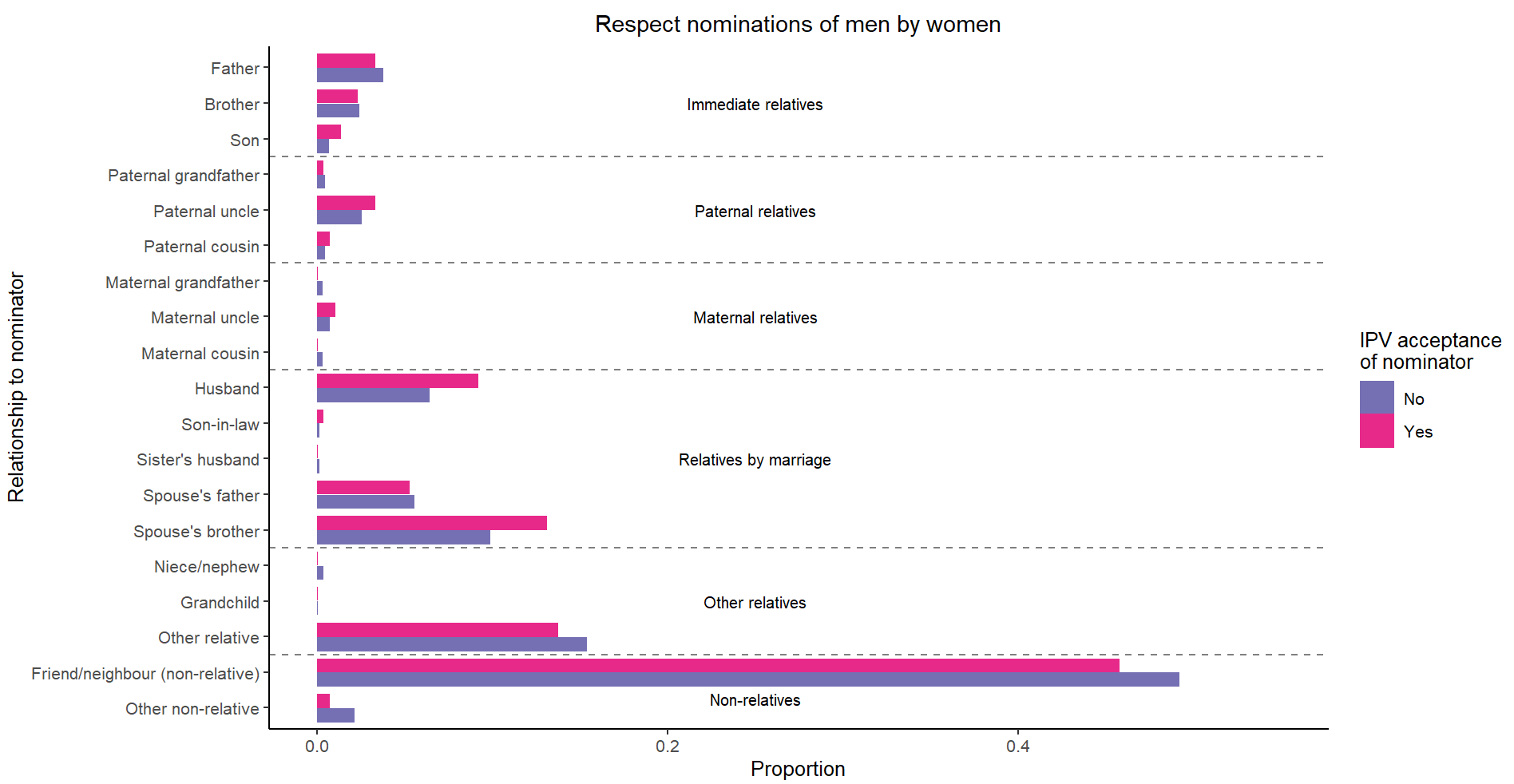


**Figure S35** Posterior distributions from *post hoc* ALAAM models estimating contagion of IPVAW-acceptance among those who reported networks. A positively skewed posterior distribution (i.e. greater than 0) indicates an individual is more likely to report accepting IPVAW if they are socially connected to another person who also accepts IPVAW. Models 1a and 6a model direct contagion among outgoing ties (as in the main model); models 1b and 6b model incoming ties (i.e. the transpose of the original matrix); models 2 and 7 additionally estimate reciprocal contagion; models 3 and 8 additionally estimate indirect contagion; models 4 and 9 additionally estimate closed indirect contagion, and; models 5 and 10 additionally estimate transitive contagion. Numbers on the right of the plot indicate the percentage of the posterior for the additional form of contagion in favour of positive contagion.


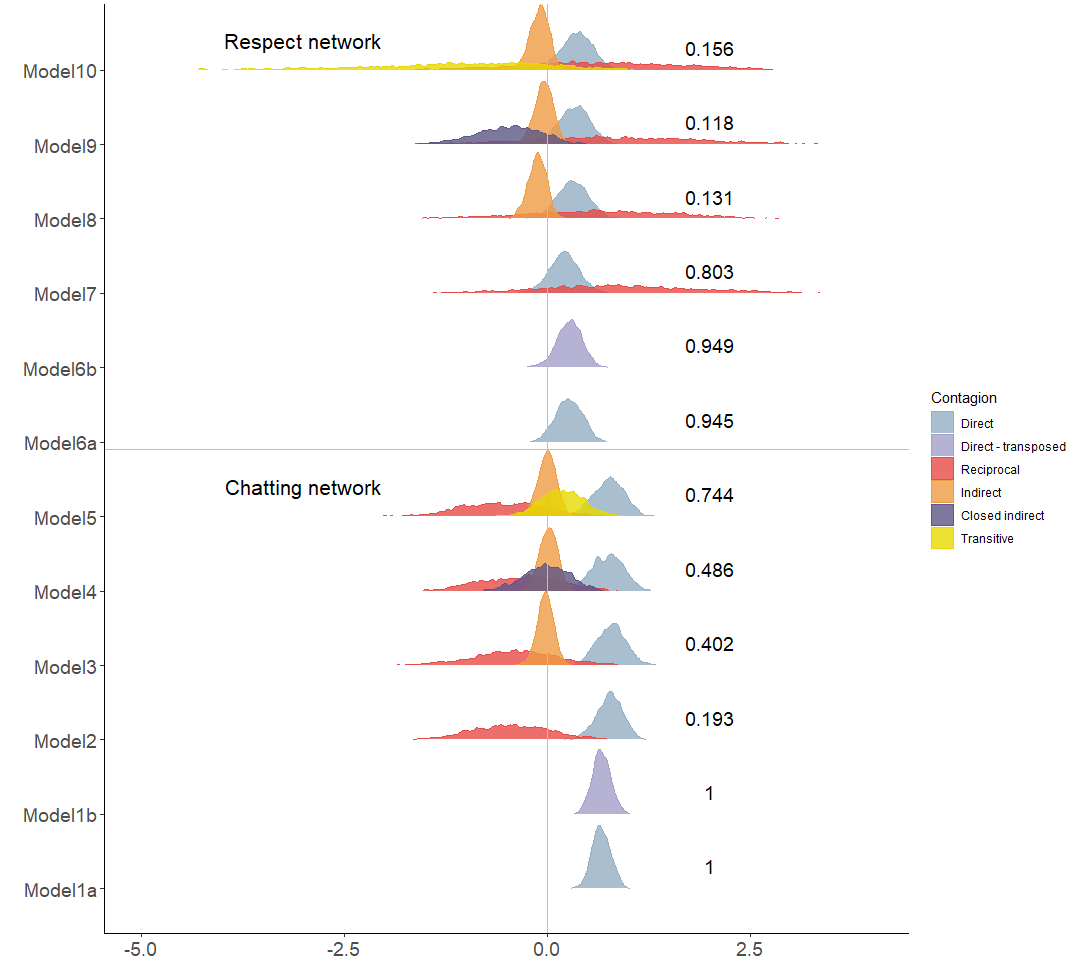


**Figure S36** Directed acyclic graph of the inferred causal relationships between variables thought relevant to the development of IPVAW acceptance. Here the outcome is IPVAW (non)acceptance of a focal individual while the exposure is the IPVAW (non)acceptance of ties (i.e. alters) in either the chatting or respect networks, resulting in the minimally sufficient adjustment set of individual-level attributes for estimating the total effect: out-degree, in-degree, education, gender, self-ranked household wealth and status, and village. Note “Unmeasured cultural factors” is a proxy for unmeasured causal factors. Empirical expectations re. men and women refer to the perceived percentage of IPV acceptance among village members, while FGMC preference refers to whether female genital mutilation/cutting if preferred for their hypothetical daughter.


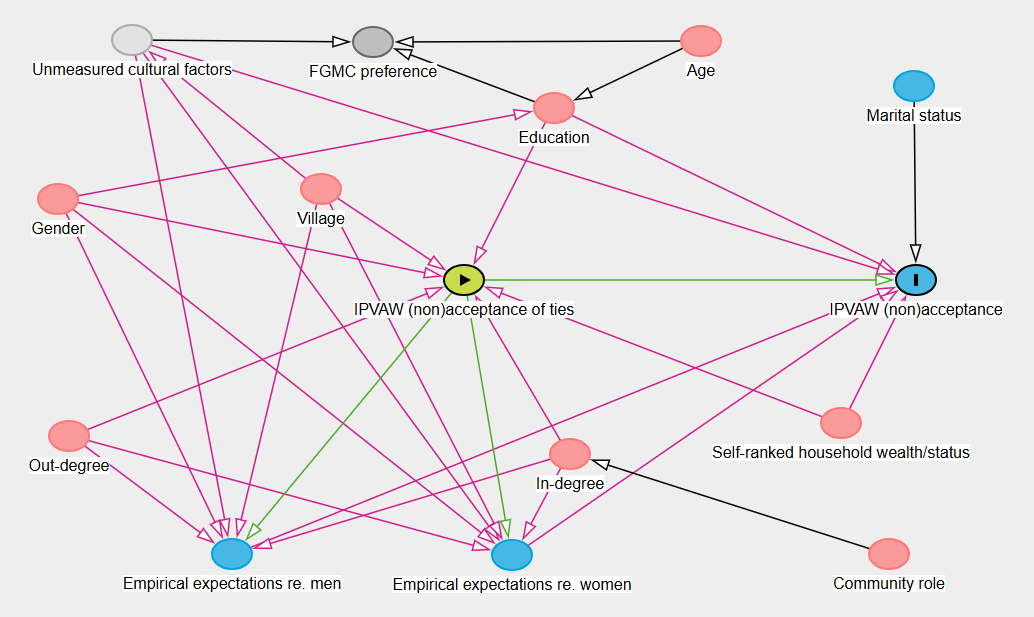


**Figure S37** Directed acyclic graph of the inferred causal relationships between variables thought relevant to the development of IPVAW acceptance. Here the outcome is IPVAW (non)acceptance of a focal individual while the exposure is the IPVAW (non)acceptance of ties (i.e. household members), resulting in the minimally sufficient adjustment set of individual-level attributes for estimating the total effect: household size (i.e. out-degree), education, gender, self-ranked household wealth and status, and village. Note “Unmeasured cultural factors” is a proxy for unmeasured causal factors. Empirical expectations re. men and women refer to the perceived percentage of IPV acceptance among village members, while FGMC preference refers to whether female genital mutilation/cutting if preferred for their hypothetical daughter.


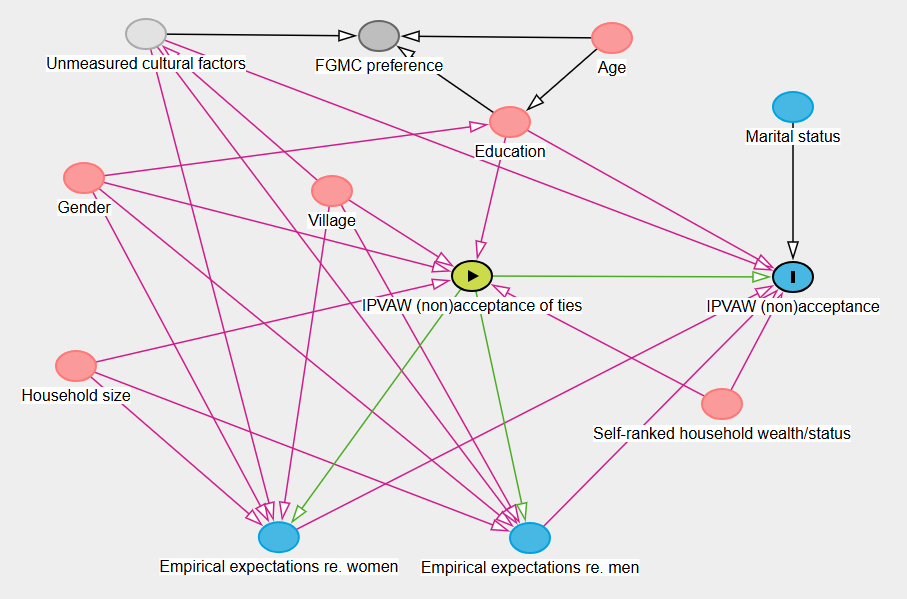

Supplement: pgaf282_Supplementary_Data [file pgaf282_supplementary_data.zip › Supplementary information file 1 PNASNEXUS-2025-00284_R2.docx]
