# Supplementary material for: Gender-biased clustering of attitudes towards physical intimate partner violence: A social network analysis in south-central Ethiopia
Source: PNAS Nexus. 2025 Sep 3;4(9):pgaf282. doi: 10.1093/pnasnexus/pgaf282 (PMC12448887; doi:10.1093/pnasnexus/pgaf282)
Supplement: pgaf282_Supplementary_Data [file pgaf282_supplementary_data.zip › SI file 3_Survey.pdf]

Questionnaire April 2021

MASTER COPY

General information

This document is for training purposes only. Please study this document carefully **before** you go out into the field. The survey documents that you (the interviewer) actually take to the field are provided separately, and are shorter and simpler.

Each survey document that you take to the field will be labelled 1A, 2A, 1B, or 2B. You will be given an equal number of copies of each. The A documents (1A and 2A) include an extra section, and will therefore take longer to administer than the B documents.

The survey documents will be given to you in pile in a specific order (1A, 1B, 2A, 2B, 1A, 1B, 2A, 2B etc) please leave them in this order. Administer them to the participants by starting at the top of the pile and then moving on to the next one.

How to follow the instructions in this document

There are different types of information in this document. They are written in different ways, as shown below.

[Some sentences are just for the interviewer's information. They are written in brackets like this sentence. These are not designed to be read out loud – they are just for your interest or for training purposes].

Some sentences contain information for the interviewer to communicate to the participant. They are written in bold letters like this. It is recommended that the interviewer memorises this content before starting fieldwork, rather than reading out each question with the exact wording. This will help them to communicate more naturally with the participant.

- ➢ Some sentences contain instructions for the interviewer to perform an action. They are shown with an arrow bullet, like this sentence. When you see a bullet point like this, it means you have to do something, such as shuffling some cards, or recording an answer in a table.

If there are multiple options for responses to the questionnaire, the interview should circle the appropriate response e.g.

(Yes = 1, No = 0)

Recording ID codes

- Before the interview starts, the Household_ID and Person_ID must be noted on the questionnaire. ID numbers will have been assigned during the Household census (conducted before the questionnaires). It is very important that the same Household_ID and Person_ID assigned during the census is used in the questionnaire.

Household_ID (4 digit)

Person_ID (6 digit)

Consent

- The interviewer starts the survey by checking whether the participant consents to be interviewed.

This is a questionnaire about your beliefs and attitudes. We hope to learn about changes in the cultural life of this village, how people experience and perceive the future, including the opportunities for marriage, education and family life. We expect that it will take around 1 hour or less to complete, but some people may take longer than this. At the end of the questionnaire you will receive a bag of coffee as a gesture of thanks for your time.

During the questionnaire we will also ask you for some details about yourself and your household. We will also ask you to imagine some events and think about your desires for your daughters and your sons wives. We If there are any question you don't want to answer, you can just ask to skip it, and you can stop at any point, without giving a reason. Only the researchers on this project with know your answers - we won't tell anyone else.

Are you happy to take part in this research?

- If the interviewer is satisfied that the participant understands and is happy to take part, they can record this in the answer box below, and proceed. Write 1 for yes, and 0 for no. If no, the interview should be terminated.

Did the participant give consent? (1=Yes, 0=No)

Introduction to participant

Thank you for agreeing to take part in this survey.

- If using Version 1A or 2A;

This survey is in four sections;

Section 1 contains some questions about you and your household
Section 2 contains some questions about the people you know and talk to
Section 3 contains some questions about preferences that people might have for their daughters, and their sons' wives
Section 4 asks some further background questions

If you would like to take a break at any point, please just say.

- If using Version 1B or 2B;

This survey is in three sections;

Section 1 contains some questions about you and your household
Section 2 contains some questions about preferences that people might have for their daughters, and their sons' wives
Section 3 asks some further background questions

If you would like to take a break at any point, please just say.

SECTION 1 PARTICIPANT DETAILS

[You should already have some of this information from the HH census. The participant's full name, the head of household's full name, relationship to the head of household, age in years, and number of completed year's education. As you fill in these details you can compare it to the names provided in the census and check that all three names match.]

OK, to start, please answer the following questions:

1. What is your full name? First name, father's name and grandfather's name?

1.1 First name ____

1.2 Father's name____

1.3 Grandfather's name ____

2. What is your head of household's first name, father's name and grandfather's name?

2.1 First name ____

2.2 Father's name____

2.3 Grandfather's name ____

3. Is this a female headed house? (Yes = 1, No = 0)

- For question 4 please use the HHH codes on the laminated sheet

4. What is your relationship to head of household? * ________________________

5. What is your age? __________ years

[For Question 6 it may be considered rude to ask the participant their sex so you can circle the relevant answer and only ask if really necessary]

6. What is your sex? (1=Male, 2= Female)

7. What is your religion? (1 = Muslim, 2= Orthodox, 3 = Protestant, 4= Waqeffatta,

5 = Other, please specify____)

8. How many years education have you completed? __________ years

9. What is your marital status today? (1= Married, 2= Never married, 3=Divorced or separated, 4= Widowed 5= Other, please specify______________________________)

- If never married, go to Q12

10. What was your age at first marriage? _________________years

11. What was your husband's / wife's age at this marriage? _______________years

12. Is this a polygamous household? (Yes = 1, No = 0)

13. Do you perform any of the following roles in the community? 1 = Teacher, 2 = Traditional Birthing Assistant, 3 = Peasant Association Leader, 4 = Militia, 5 = Religious leader, 6 = None, 7 = Other, please specify_________________

14. Were you born in this zone? (Yes = 1, No = 0)

- If Yes go to Qu 18

15. Please state the name of your birth zone, and birth kebele

15.1 Birth zone ______________________________________

15.2 Birth kebele______________________________________

16. Why did you move? (1= For marriage, 2= For work, 3 = As a child with parents, 4= Other, please specify____)

17. How many years have you lived in this zone? __________________Years

18. How many children did you (or your wife/wives) give birth to?

[The aim of this question is to understand how many children the participant has given birth to (or their wives have given birth to, including children who have died. It may be easiest to ask questions 18.1 – 18.6 in the order shown in the table below, although it doesn't really matter. Importantly, once questions 18.1 – 18.6 are completed, 18.7 is completed to confirm the total number of births of all children alive and dead, which should be a sum of all the births mentioned in 18.1 – 18.6.]

18.1	18.2	18.3	18.4	18.5	18.6	18.7

Boys alive < age 15 today	Boys who survived > age 15	Boys who died before age 15	Girls alive < age 15 today	Girls who survived > age 15	Girls who died before age 15	So total births

19. Have any of your children got married?

19.1 Sons (Yes = 1, No = 0)

19.2 Daughters (Yes = 1, No = 0)

Now please can you tell us about your parents;

	**Mother**	**Father**
**Still alive?**	20. No Yes	23. No Yes
**First name**	21.1	24.1
**Father's name**	21.2	24.2
**Grandfather's name**	21.3	24.3
**Place of residence (circle):**	22.1. Same zone<br>22.2. Other zone (specify):<br>22.3. Other kebele	25.1. Same zone<br>25.2. Other zone (specify):<br>25.3. Other kebele

End of Section 1

SECTION 2 SOCIAL NETWORKS

***** Section 2 is only included for Questionnaire versions 1A and 2A *****

[The aim of the questions in this section is to find out who the participant interacts with in a number of different circumstances.

- It is very important that we are able to match the people listed by the participant in response to these questions, to the individuals in all the household census. For this reason we are asking for their name, father's name, and grandfather's name, as well as other identifying characteristics such as age and residence. It is important therefore that the names are recorded carefully. Together this should provide enough information so that the named individuals and their Individual_ID can be identified in the household census. (Obviously this will not be possible where people named by respondent do not live in one of the sample zones).
- There is space for 10 people to be named in answer to each question, but the table does not have to be filled for all questions. We expect most participants will name between 2-8 people in answer to each question.]

- ➢ The interview must be conducted in private. Confirm that there is no one else in the room or who can overhear the interview.
- ➢ Read the general blurb.
- ➢ Ask Qu 2.1 (read out the question, and the explanation after it). Write the full names of the people named by the participant (their name, father's name, and grandfather's name).
- ➢ When the participant has named all the people they can think of in response to Qu 2.1, ask "**Is there anyone else**?" and repeat this until the participant cannot think of anyone else.
- ➢ Before filling in the rest of the table for Qu 2.1, go to Qu 2.2 and repeat the process, and so on up until Qu 2.6
- ➢ If the participant doesn't want to answer a question this can be recorded at the top of the sheet (DWTA)
- ➢ Once all the names have been generated for Qu 2.1 – 2.6 go back to Qu 2.1 and fill in the details for the people who have been named, i.e. their location, sex, age, occupation and relationship to the participant (using the codes as appropriate).
- ➢ The Person_ID code can be completed later, by matching the details of the people named to the HH census.

GENERAL BLURB

In our life, each of us, in different ways, interact with other people for advice, support, money and conversation. To get a sense of who you interact with in different parts of your life, we will ask you a number of questions and ask you to name those people who you interact with in different ways.

The same people can be named in answer to the different questions, but they must be 15 years or older.

<u>Name generator questions:</u>

2.1 Who do you spend time chatting with? *This could be inside the home or when you're out and about, this could be when you're doing your chores or working, when eating a meal, or walking somewhere. Please give the names of anyone that you spend time chatting with no matter what you're doing. They could be friends, family members, people you work with, or neighbours etc.*

2.2 Who would you go to for advice on preparing your daughter for marriage? Or if you do not have a daughter, who would you go to if you did have a daughter. *This could be for advice about a marriage partner, or about dowry or bridewealth arrangements, or anything else to do with preparing a daughter for marriage. Please give the names of the people you would go to for this type of advice. They could be friends, family members, people you work with, or neighbours etc.*

2.3 Who would come to you for advice on preparing their daughter for marriage? *This could be advice about a marriage partner, or about dowry or bridewealth arrangements, or anything else to do with preparing a daughter for marriage. Please give the names of the people who would come to you for this type of advice. They could be friends, family members, people you work with, or neighbours etc*

2.4 Who do you respect and admire? *These could be people you talk to regularly, or they could be people you do not know well or talk to, but their opinions are important to you. They could be friends, family members, people you work with, or neighbours etc.*

2.5 From whom would you feel comfortable asking to borrow 100 birr if you needed it? *Sometimes, for example in an emergency, it might be necessary to borrow money from someone. Please give the names of people you would feel comfortable borrowing 100 birr. They could be friends, family members, people you work with, or neighbours etc.*

2.6 Who do you think would be comfortable asking to borrow 100 birr from you if they needed it? *Sometimes, for example in an emergency, someone might need to borrow money from you. Please give the names of people you think would feel comfortable asking to borrow 100 birr from you. They could be friends, family members, people you work with, or neighbours etc.*

2.1 Who do you spend time chatting with? ***DWTA*** ☐

	**Person 1**	**Person 2**	**Person 3**	**Person 4**	**Person 5**
**First name**					
**Father's name**					
**Grandfather's name**					
**Place of residence (circle):**	1. Same zone<br>2. Other zone (specify):	1. Same zone<br>2. Other zone (specify):	1. Same zone<br>2. Other zone (specify):	1. Same zone<br>2. Other zone (specify):	1. Same zone<br>2. Other zone (specify):
**Sex**					
**Age**					
**Relationship to you**					
**Person_ID code**					

	**Person 6**	**Person 7**	**Person 8**	**Person 9**	**Person 10**
**First name**					
**Father's name**					
**Grandfather's name**					
**Place of residence (circle):**	1. Same zone<br>2. Other zone (specify):	1. Same zone<br>2. Other zone (specify):	1. Same zone<br>2. Other zone (specify):	1. Same zone<br>2. Other zone (specify):	1. Same zone<br>2. Other zone (specify):
**Sex**					
**Age**					
**Relationship to you**					
**Person_ID code**					

2.2 Who would you go to for advice on preparing your daughter for marriage?
Or, if you do not have a daughter, who would you go to if you did have a daughter. ***DWTA*** ☐

	**Person 1**	**Person 2**	**Person 3**	**Person 4**	**Person 5**
**First name**					
**Father's name**					
**Grandfather's name**					
**Place of residence (circle):**	1. Same zone<br>2. Other zone (specify):	1. Same zone<br>2. Other zone (specify):	1. Same zone<br>2. Other zone (specify):	1. Same zone<br>2. Other zone (specify):	1. Same zone<br>2. Other zone (specify):
**Sex**					
**Age**					
**Relationship to you**					
**Person_ID code**					

	**Person 6**	**Person 7**	**Person 8**	**Person 9**	**Person 10**
**First name**					
**Father's name**					
**Grandfather's name**					
**Place of residence (circle):**	1. Same zone<br>2. Other zone (specify):	1. Same zone<br>2. Other zone (specify):	1. Same zone<br>2. Other zone (specify):	1. Same zone<br>2. Other zone (specify):	1. Same zone<br>2. Other zone (specify):
**Sex**					
**Age**					
**Relationship to you**					
**Person_ID code**					

2.3

2.4 Who would come to you for advice on preparing their daughter for marriage? ***DWTA*** ☐

	**Person 1**	**Person 2**	**Person 3**	**Person 4**	**Person 5**
**First name**					
**Father's name**					
**Grandfather's name**					
**Place of residence (circle):**	1. Same zone<br>2. Other zone (specify):	1. Same zone<br>2. Other zone (specify):	1. Same zone<br>2. Other zone (specify):	1. Same zone<br>2. Other zone (specify):	1. Same zone<br>2. Other zone (specify):
**Sex**					
**Age**					
**Relationship to you**					
**Person_ID code**					

	**Person 6**	**Person 7**	**Person 8**	**Person 9**	**Person 10**
**First name**					
**Father's name**					
**Grandfather's name**					
**Place of residence (circle):**	1. Same zone<br>2. Other zone (specify):	1. Same zone<br>2. Other zone (specify):	1. Same zone<br>2. Other zone (specify):	1. Same zone<br>2. Other zone (specify):	1. Same zone<br>2. Other zone (specify):
**Sex**					
**Age**					
**Relationship to you**					
**Person_ID code**					

2.5 Who do you respect and admire? *DWTA* ☐

	**Person 1**	**Person 2**	**Person 3**	**Person 4**	**Person 5**
**First name**					
**Father's name**					
**Grandfather's name**					
**Place of residence (circle):**	1. Same zone<br>2. Other zone (specify):	1. Same zone<br>2. Other zone (specify):	1. Same zone<br>2. Other zone (specify):	1. Same zone<br>2. Other zone (specify):	1. Same zone<br>2. Other zone (specify):
**Sex**					
**Age**					
**Relationship to you**					
**Person_ID code**					

	**Person 6**	**Person 7**	**Person 8**	**Person 9**	**Person 10**
**First name**					
**Father's name**					
**Grandfather's name**					
**Place of residence (circle):**	1. Same zone<br>2. Other zone (specify):	1. Same zone<br>2. Other zone (specify):	1. Same zone<br>2. Other zone (specify):	1. Same zone<br>2. Other zone (specify):	1. Same zone<br>2. Other zone (specify):
**Sex**					
**Age**					
**Relationship to you**					
**Person_ID code**					

2.6 From whom would you feel comfortable asking to borrow 100 birr if you needed it? ***DWTA*** ☐

	**Person 1**	**Person 2**	**Person 3**	**Person 4**	**Person 5**
**First name**					
**Father's name**					
**Grandfather's name**					
**Place of residence (circle):**	1. Same zone<br>2. Other zone (specify):	1. Same zone<br>2. Other zone (specify):	1. Same zone<br>2. Other zone (specify):	1. Same zone<br>2. Other zone (specify):	1. Same zone<br>2. Other zone (specify):
**Sex**					
**Age**					
**Relationship to you**					
**Person_ID code**					

	**Person 6**	**Person 7**	**Person 8**	**Person 9**	**Person 10**
**First name**					
**Father's name**					
**Grandfather's name**					
**Place of residence (circle):**	1. Same zone<br>2. Other zone (specify):	1. Same zone<br>2. Other zone (specify):	1. Same zone<br>2. Other zone (specify):	1. Same zone<br>2. Other zone (specify):	1. Same zone<br>2. Other zone (specify):
**Sex**					
**Age**					
**Relationship to you**					
**Person_ID code**					

2.7 Who do you think would be comfortable asking to borrow 100 birr from you if they needed it? ***DWTA*** ☐

	**Person 1**	**Person 2**	**Person 3**	**Person 4**	**Person 5**
**First name**					
**Father’s name**					
**Grandfather’s name**					
**Place of residence (circle):**	1. Same zone<br>2. Other zone (specify):	1. Same zone<br>2. Other zone (specify):	1. Same zone<br>2. Other zone (specify):	1. Same zone<br>2. Other zone (specify):	1. Same zone<br>2. Other zone (specify):
**Sex**					
**Age**					
**Relationship to you**					
**Person_ID code**					

	**Person 6**	**Person 7**	**Person 8**	**Person 9**	**Person 10**
**First name**					
**Father’s name**					
**Grandfather’s name**					
**Place of residence (circle):**	1. Same zone<br>2. Other zone (specify):	1. Same zone<br>2. Other zone (specify):	1. Same zone<br>2. Other zone (specify):	1. Same zone<br>2. Other zone (specify):	1. Same zone<br>2. Other zone (specify):
**Sex**					
**Age**					
**Relationship to you**					
**Person_ID code**					

SECTION 3 ATTITUDES TO FAMILY

**** Survey 1A or 1B only ****

Now we are going to ask you some questions about your desires for your family.

- In this version of Section 3, you just show 4 cards to the respondent in the first part keeping the FGM card hidden, and then in the second part all 5 cards are used.

- First take out only the 4 non-sensitive cards. Then read the following to the participant;

I'd like you to imagine that you are the parent of a girl who has not yet become an adult. Family have wishes and aims for their children. There are some things that they hope will happen to them by the time they are adults, or which they will try to ensure happens if possible. There are other things that they do not particularly want or do not care about.

Here I have some cards. On each card there is a picture of something that you might (or might not) want for your daughter by the time she is an adult.

Please don't tell me which of the things on these cards you personally would want for your daughter. Instead, I'd like you to tell me <u>how many</u> of these cards show things that you want for your daughter.

It's important that you don't tell me which individual things you are choosing, just give me a number. You can choose as many or as few as you like. If you'd like to hold or move the cards that is fine, but please don't tell me which particular cards you are choosing.

Qu 3.1 How many of these would you want for your daughter?

- Hand the four cards to the respondent to look at, with early marriage, work in the city, go to college, live close to home

**Daughter:** How many cards were selected?	

OK, we're going to repeat that a second time. This time, I'd like you to imagine that you are the parent of a man who is soon going to be married. I'm going to read out those things again, and this time, please think about which of these things you would want your son's wife to have. Your preferences might be the same as before, or a bit different this time. Either of these is fine; please just be honest.

Qu 3.2 How many of these would you want for your son's wife?

- Hand the four cards to the respondent to look at, with early marriage, work in the city, go to college, live close to home

**Son's wife:** How many cards were selected?	

- Go to Qu 3.5

**** Survey 2A or 2B only ****

- Take out all 5 cards, including the FGM card. Then read the following to the participant;

I'd like you to imagine that you are the parent of a girl who has not yet become an adult, and consider the things that you would or would not want for your daughter. Family have wishes and aims for their children. There are some things that they hope will happen to them by the time they are adults, or which they will try to ensure happens if possible. There are other things that they do not particularly want or do not care about.

Here I have some cards. On each card there is a picture of something that you might (or might not) want for your daughter by the time she is an adult.

Please don't tell me which of the things on these cards you personally would want for your daughter. Instead, I'd like you to tell me how many of these cards show things that you want for your daughter.

It's important that you don't tell me which individual things you are choosing, just give me a number. You can choose as many or as few as you like. If you'd like to hold or move the cards that is fine, but please don't tell me which particular cards you are choosing.

Qu 3.1 How many of these would you want for your daughter?

- Hand the five cards to the respondent to look at, with FGM, early marriage, work in the city, go to college, live close to home

**Daughter:** How many cards were selected?	

OK, as before we're going to repeat that a second time. This time, I'd like you to imagine that you are the parent of a man who is soon going to be married. I'm going to read out those things again, and this time, please think about which of these things you would want your son's wife to have. Your preferences might be the same as for your daughter, or a bit different this time. Either of these is fine; please just be honest.

Qu 3.2 How many of these would you want for your son's wife?

- Hand the five cards to the respondent to look at, with FGM, early marriage, work in the city, go to college, live close to home

**Son's wife:** How many cards were selected?	

- Go to Qu 3.5

[Question 3.5 is the same for all versions of the questionnaire. In this last question instead of asking the number of items they would like, you will ask the participant directly which of the items they would like for their daughter, and then their son's wife]

Qu 3.5 We are going to look at each of the cards one last time.

- Now take all cards out, so that all five cards are present including the FGM card.

This time I'd like you to tell me whether you would want any of these for a daughter.

- Go through each card asking 'would you want this for your daughter?' and mark their response.

Would you want this for a daughter? (1=Yes, 0=No)				
Early marriage	Work in the city	Go to college	FGM	Live close to home

Qu 3.6 And finally, which of these would you want for your son's wife?

- Go through each card asking 'would you want this for your son's wife?' and mark their response.

Would you want this for your **son's wife**? (1=Yes, 0=No)				
Early marriage	Work in the city	Go to college	FGM	To live close to home

SECTION 4 BACKGROUND DETAILS

[The final section is on a mixture of topics. Some questions are about the participant's attitudes towards issues affecting women and girls, as well as about their opinion of the views of their community towards these issues. The other questions are about their living circumstances, e.g. their wealth and access to media]

[Question 4.1 below asks "what type of marriage would you like for a daughter". For participants who do not have a daughter themselves, you can ask them what they would like if they did have a daughter. Similarly for Question 4.2 and 4.3 if the participant does not have children they should answer as if they did have children]

Now we would like to ask you some further questions. Some are questions about issues which affect the lives of women and girls in this community, other questions are about your living circumstances.

4.1 Some marriages are arranged by parents, whereas some young men and women choose their own marriage partners in a love marriage. What type of marriage would you like for a daughter?

(1= brideprice, 2 = abduction, 3 = exchange, 4 = levirate, 5 = sister exchange,

6 = "love marriage"/ not arranged , 7 = don't mind)

The next questions are about the ideal age of marriage for your children, no matter what type of marriage it is.

4.2 In your view, what would be the ideal age of first marriage for a daughter_________ yrs

4.3 And likewise, what would be the ideal age of first marriage for a son?_____________yrs

[In **Questions 4.4**, **4.5**, **4.7** and **4.8** the aim is to find out what people think their fellow villagers think about the acceptability of both FGC and wife beating. In other words, in the participant's view, what proportion of people in the village either want FGC for their daughter, or think that wife beating is acceptable?

Some participants may not might not give a percentage, and their responses will need to be interpreted as percentages such as **none** (0%), **half** (50%), **all** (100%). If it is unclear, you may need to be probe to get a more accurate response. For example if their answer is **not many** you could find out if they mean 10% or 20% or 30% using the scale below as a visual aid.

Circle the relevant percentage on the scale drawing.]

In a previous question we asked about several life events that you might want for a daughter or daughter-in-law, including FGC. We are interested to know what the other people who live in your zone think about FGC.

4.4 Can you indicate on this line how many <u>men</u> in your zone you think would want FGC for their daughter?

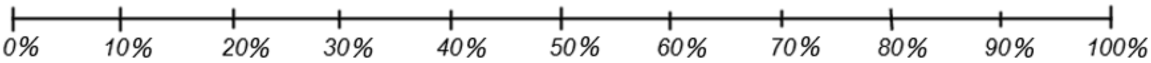

4.5 Now please can you indicate on this line how many <u>women</u> in your zone you think would want FGC for their daughter?

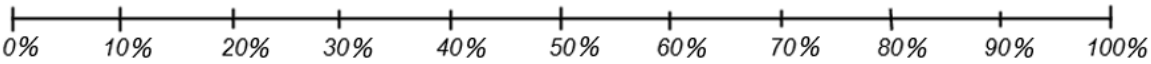

4.6 Finally, if a family in this zone arranged FGM for their daughter, do you think other people in the zone would; [circle one answer]

a) Approve of their action

b) Disapprove of their action

c) Think it was none of their business

4.7 Now I'd like to ask you about men's attitudes toward their wives. Do you think it is acceptable sometimes for a husband to beat his wife?

[If the participant agrees that it is sometimes acceptable for a husband to beat his wife, you then ask under what circumstances. Participants can give up to three different circumstances, but are not required to give three different circumstances. It is fine if they only come up with one or two. Please be as accurate as you can in recording their answers]

Sometimes acceptable for a husband to beat his wife? (1=Yes, 0=No)	If yes, under what circumstances?<br><br>[Ask respondents to give up to three circumstances]

We are also interested to know your views of what other people in your zone think about men beating their wives.

4.7 Can you indicate on this line how many men in your zone you think would find it acceptable sometimes for a husband to beat his wife?

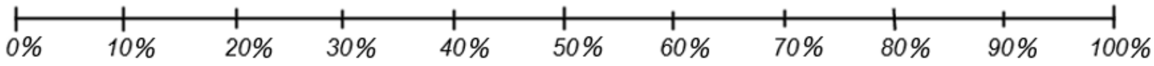

4.8 Can you indicate on this line how many of the women in your zone you think would find it acceptable sometimes for a husband to beat his wife?

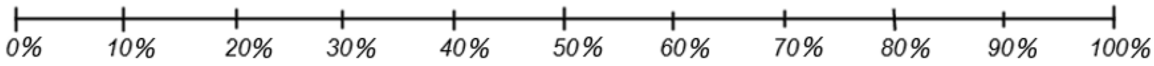

The next questions are about family size, and the number of children a person would desire.

[For questions 4.9, 4.10 or 4.11 please insist that the respondent gives a numerical answer. For example if they say 'as many as I'm blessed with' please probe to find a numerical answer for the number of children they personally think would be ideal.

For respondents who have some children already, this question is not asking how many more children they would like. The aim of the question is to find out their ideal number of children to have over their lifetime if they could choose now].

4.9 If you could choose exactly the number of children to have in your whole life, what would be your ideal number of children?___________________

We are also interested to know your views about other peoples' ideal number of children.

4.10 Please can you tell me the ideal number of children you think most men in this zone would have if they could choose?________________________________

4.11 Please can you tell me the ideal number of children you think most women in this zone would have if they could choose?________________________________

4.12 *In a previous question we asked about men's attitudes towards their wives. Now I'd like to ask you about women's attitudes toward their husbands. Do you think it is acceptable sometimes for a wife to beat her husband?*

[If the participant agrees that it is sometimes acceptable for a wife to beat her husband, you then ask under what circumstances. Participants can give up to three different circumstances, but are not required to give three different circumstances. It is fine if they only come up with one or two. Please be as accurate as you can in recording their answers]

Sometimes acceptable for a wife to beat her husband? (1=Yes, 0=No)	If yes, under what circumstances?<br><br>[Ask respondents to give up to three circumstances]

The next questions are about wealth and status. I would like to know about how you see your position in your village.

5.1 ***Please look at the pictures of 10 houses shown here. These houses represent the 10 different levels of wealth and status of the households in your village. The richest tenth are represented by the [large] house on the far left and the poorest tenth by the [small] house on the right.***

I'd like you to use this picture to tell me what you think your position is. If you think you are in the top tenth, choose the house on the very left. If you think you are in the bottom tenth, choose the one on the right. Or you can choose any house in between. Please pick a house" [interviewer circle the relevant number]

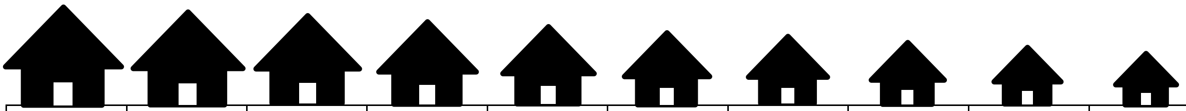

1	2	3	4	5	6	7	8	9	10

5.2 ***Do you and your head of household own any land?***

You own land? (1=Yes, 0=No)	Head of household owns land? (1=Yes, 0=No)

5.3 ***Do you own a ………..? Does your head of household own a…..?***

- ***Working radio***
- ***Working mobile phone***
- ***Working television***

Do you own…? (1=Yes, 0=No)		
Radio	Mobile	Television

Does your head of household own…? (1=Yes, 0=No)		
Radio	Mobile	Television

5.4 Media exposure

[For each of the questions below, the interviewer should select the number from the list below that comes closest to the participant's answer, and write it in the answer sheet. So, for example, if the participant says "about once a week", write "3". The interviewer does not need to read out the options unless desired, but can use them to confirm (e.g. "so, are you saying about once a week?). There is no need to read the actual number (i.e. 1,2,3,4) to the participant, as this may be confusing to them.]

Typically:
How often do you listen to the radio?
How often do you watch TV (television)?
How often do you access social media or internet (e.g. YouTube)

1. Never
2. Rarely (once or twice a month)
3. Sometimes (about once or twice a week)
4. Often (every day, or almost every day)

How often do you…? (write 1,2,3 or 4)		
Listen to radio	Watch TV	Internet

If you have been affected by any of the issues in this questionnaire, please talk to the enumerators who can direct you to access local support.

That is the end of the interview. As promised, here is a bag of coffee to say Thank You for your time.

End of interview
